# The Effects of General Anaesthesia and Light on Behavioural Rhythms and GABA_A_ Receptor Subunit Expression in the Mouse SCN

**DOI:** 10.3390/clockssleep3030034

**Published:** 2021-09-17

**Authors:** Janelle Chong, James Frederick Cheeseman, Matthew D. M. Pawley, Andrea Kwakowsky, Guy R. Warman

**Affiliations:** 1 Department of Anaesthesiology, School of Medicine, University of Auckland, Auckland 1142, New Zealand; janelle.chong@auckland.ac.nz (J.C.); j.cheeseman@auckland.ac.nz (J.F.C.); m.pawley@massey.ac.nz (M.D.M.P.); 2 School of Natural and Computational Sciences, Massey University, Auckland 0745, New Zealand; 3 Department of Anatomy and Medical Imaging, School of Medical Sciences, University of Auckland, Auckland 1142, New Zealand; a.kwakowsky@auckland.ac.nz

**Keywords:** general anaesthesia, circadian clock, phase response curve, GABA, GABA_A_ receptor

## Abstract

General anaesthesia (GA) is known to affect the circadian clock. However, the mechanisms that underlie GA-induced shifting of the clock are less well understood. Activation of γ-aminobutyric acid (GABA)_-_type A receptors (GABA_A_R) in the suprachiasmatic nucleus (SCN) can phase shift the clock and thus GABA and its receptors represent a putative pathway via which GA exerts its effect on the clock. Here, we investigated the concurrent effects of the inhalational anaesthetic, isoflurane, and light, on mouse behavioural locomotor rhythms and on α1, β3, and γ2 GABA_A_R subunit expression in the SCN of the mouse brain. Behavioural phase shifts elicited by exposure of mice to four hours of GA (2% isoflurane) and light (400 lux) (*n* = 60) were determined by recording running wheel activity rhythms in constant conditions (DD). Full phase response curves for the effects of GA + light on behavioural rhythms show that phase shifts persist in anaesthetized mice exposed to light. Daily variation was detected in all three GABA_A_R subunits in LD 12:12. The γ2 subunit expression was significantly increased following GA in DD (compared to light alone) at times of large behavioural phase delays. We conclude that the phase shifting effect of light on the mouse clock is not blocked by GA administration, and that γ2 may potentially be involved in the phase shifting effect of GA on the clock. Further analysis of GABA_A_R subunit expression in the SCN will be necessary to confirm its role.

## 1. Introduction

General anaesthesia (GA) is administered to well over a hundred million patients annually worldwide, and is an essential part of most major operations [1]. The safety and efficacy of anaesthetics have consistently improved since its inception, with anaesthesia-related mortality dropping from 64 per million in the 1940s to 4 per million in the last decade [2]. There are, however, side effects of GA and one of these appears to be an effect on postoperative sleep and circadian rhythms [3]. Postoperative sleep disturbances are common in surgical patients and are known to have adverse effects on patient recovery [4,5]. Evidence has shown that GA can shift the circadian clock and this may directly contribute to the development of postoperative sleep disturbances [6]. The overarching goal of our work is to understand how GA affects the clock, and to find ways in which GA-induced clock shifts can be ameliorated. In order to achieve this goal, a thorough investigation of the effects of GA on the clock, the interaction of GA with known *zeitgeber* (such as light), and mechanisms that underlie GA-induced shifting of the clock is essential.

Our group have made inroads into the investigation of the effects of GA on the circadian clock in a range of model (invertebrate and vertebrate) organisms over the past decade [6,7]. We have shown that GA can phase shift behavioural rhythms of honeybees (*Apis mellifera*) and *Drosophila melanogaster* in a time-dependent manner [6,8], and that these behavioural shifts are underpinned by changes in the expression of core clock genes. Most recently, we have demonstrated that the inhalational agent isoflurane causes robust and reproducible shifts in behaviour and PERIOD2 (PER2) expression in mice, with maximal shifts occurring between CT6 and CT16 [9].

Light is the most potent *zeitgeber* for circadian clocks, and while its phase shifting effect on the clock is well understood, less is known about how the effects of light and anaesthesia may interact. Anaesthesia is, of course, administered in ‘real-world” conditions (rather than constant conditions), thus it is relevant to try and understand the effects of GA on the clock as they compare to those of light, and what the combined effects of light and GA on the clock might be. Our work in honey bees has demonstrated that the phase response curves for GA and light are opposing at early circadian times [7]. When administered together, light reduces GA-induced phase shifting in bees. Whether this occurs in mammals remains to be understood. There is, however, a fundamental difference in the timing of GA-induced phase shifts and their relationship to light-induced phase shifts in mammals, as the circadian times at which phase advances and delays occur with GA and light are not in antiphase (as they are in bees) with both GA and light causing delays between CT6 and CT18 in mice [9,10].

While there is a substantial body of evidence to demonstrate that GA affects behaviour and clock gene expression in a range of species, there is a paucity of information on the neuronal/neurotransmitter pathways that may mediate GA’s effect on the clock. GA has been shown to be capable of directly affecting the clock by reducing the acetylation of *E*-box regulatory regions of PER2 [11]. However, activation of neurotransmitters such as γ-aminobutyric acid (GABA) has also been shown to cause phase shifts [12].

GA is known to have strong effects on neurotransmitter systems in the brain, one of the key molecular targets being the inhibitory neurotransmitter GABA [13]. Most clinical anaesthetics, such as isoflurane and propofol, act at least in part through potentiating the effect of GABA on the GABA-type A receptor (GABA_A_R). The GABA_A_R is a pentameric structure most commonly comprised of two alpha 1 (α1) subunits, two beta 2 (β2) subunits and one gamma 2 (γ2) subunit. There are, however, at least 20 different GABA_A_R subunits which can make up different receptor configurations in different tissues [14]. The expression pattern of subunits is specific to the brain region and function [15]. The subunit composition of GABA_A_Rs has a direct effect on the efficacy and affinity of anaesthetic action on the GABAergic system [16,17,18]. Studies have shown that GABA_A_Rs containing an α and β subunits are sensitive to volatile anaesthetics, and that administration of GA results in an increase in the surface expression of GABA_A_R subunits thus potentiating the effect of GABA further [19,20,21].

GABA is also an important neurotransmitter of the circadian clock, and multiple GABA_A_R subunits have been identified throughout the SCN including α1, β3, and γ2 [22,23,24]. Although the exact role of GABA in the SCN has yet to be fully elucidated, GABAergic neurotransmission has been implicated in various circadian functions including entrainment of the clock and coupling between different SCN sub-oscillators [25,26].

Here, we describe experiments investigating the behavioural phase shifting effects of GA administered together with light, and compare them to those of light alone in order to determine the combined effects of these two different agents in a mammalian model. Furthermore, we describe the investigation of the effects of GA and light on the expression of GABA_A_R subunits in the SCN to examine whether this neurotransmitter may be involved in GA-induced clock shifting. In this work, we have chosen to look at the expression of three different subunits which are expressed in the SCN (α1, β3, γ2) as representatives of three of the major subtypes of GABA_A_R subunits (alpha, beta, and gamma) to provide an initial understanding of whether their expression is influenced by GA, and light, and whether they may mediate GA-induced phase shifting of the clock.

## 2. Results

### 2.1. Effects of GA and Light on Locomotor Rhythms of C57 Mice

The phase shifting effects of GA plus light (and light alone) were determined by administering a 4 h GA (2% isoflurane) with light (400 lux) using an Aschoff-type II protocol (see methods). Of the 120 C57BL/6 mice used for the behavioural experiments, 118 provided usable data (58 for the light group and 60 for GA + Light group) (Supplementary Table S1, Supplementary Figure S1). The two animals excluded did not display rhythmic behavioural activity prior to treatment and therefore did not provide analysable data.

**Figure 1:**
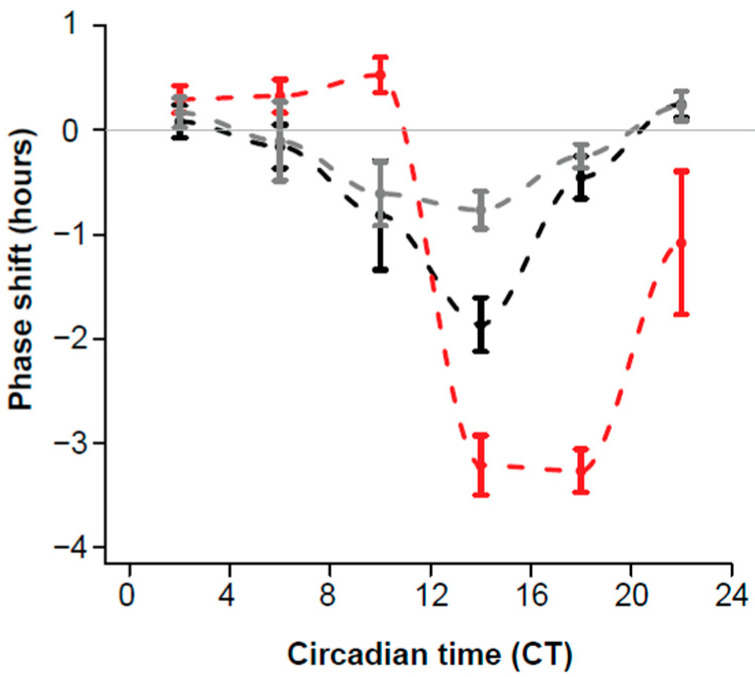
Phase response curves for the effects of four hours of isoflurane + light (black line) (*n* = 60) and 4 h of light (red line) (*n* = 58) on locomotor activity of C57/BL6 mice at different circadian times. Data are binned into four hour epochs. Error bars represent the standard error of the mean (SEM) of each epoch. Grey line at zero represents no phase shift. The PRC for 6 h of GA in DD is plotted in grey for comparison [9].

Isoflurane anaesthesia administered together with light produced robust shifts in the phase of locomotor activity rhythms in C57 mice (Figure 1, Table 1). There was no observable effect on the underlying period of the clock, with a mean free-running period of 23.83 h (±0.014) before anaesthesia and 23.70 h (±0.023) after anaesthesia.

Behavioural phase shifts elicited by concurrent isoflurane and light treatment (4 h) produced a ‘weak” or type I PRC (Figure 1, Table 1). A pronounced delay portion was observed between CT8 and CT18, with a maximum delay of –1.87 h (±0.26 h). Phase advances were generally very much smaller in magnitude, with the maximum advance of 0.25 h (±0.12 h) elicited between CT20 and CT3. The largest phase delay and phase advance of individual animals were –4.7 h at CT8 and 0.94 h at CT1.5, respectively.

The phase shifts attributable to light alone were of a magnitude and time previously reported by other authors with a maximum phase advance of 0.53 h (±0.17 h) and delay of –3.27 (±0.29 h) (Table 1), respectively [10]. For the sake of comparison, the data from behavioural phase shifts elicited by 2% isoflurane (6 h) are over-plotted. The shifting effect of GA in DD (6 h) and GA in light (4 h) is very similar in magnitude, with a more profound phase shift evident in the GA plus light condition (between CT13 and CT20).

### 2.2. GABA_A_R Subunit Expression in the SCN under LD Conditions

Two experiments were conducted in order to determine the effect of GA and light on GABA_A_R expression in the SCN of mice exposed to different treatments. The first of these was an expression profile of three different receptor subunits under standard LD 12:12 light cycles.

**Figure 2:**
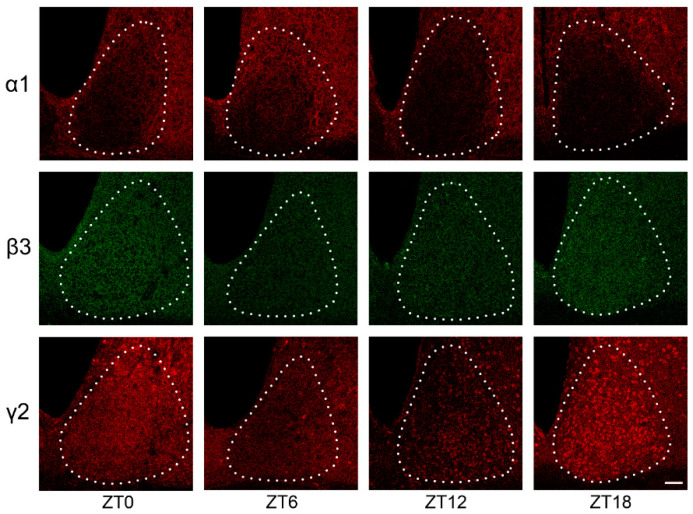
Representative photomicrographs of GABA_A_R α1, β3, and γ2 subunit immunoreactivity in the SCN across four different time points in LD 12:12. Scale bar = 50 µm. Intensity of staining is indicated by strength of red (α1, and γ2) and green (β3) signal. Margins of the SCN are indicated by dashed white line.

The GABA_A_R α1, β3, and γ2 subunits displayed immunoreactivity throughout the SCN (Figure 2) (Supplementary Table S2, Supplementary Figure S2). Protein immunoreactivity was measured and analysed across the rostrocaudal length of the mouse SCN over the course of 24 h.

**Figure 3:**
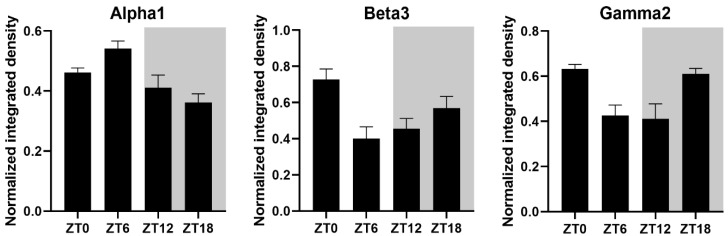
Quantification of GABA_A_R α1, β3, and γ2 subunit immunoreactivity in the mouse SCN from mice maintained in a light-dark cycle (LD 12:12). Timing of the light and dark portions of the day are indicated by the white and grey backgrounds respectively. The data are graphed as mean density of staining normalized to peak staining (as 1.0) ± SEM.

All three GABA_A_R subunits exhibited daily variation in expression under LD 12:12 (Figure 2 and Figure 3). The β3 and γ2 subunits showed a similar trend in protein expression in the SCN, with higher levels of immunoreactivity at ZT18 and ZT0 which decreased between ZT6 and ZT12.

In contrast, α1 subunit expression was in anti-phase to the β3 and γ2 subunits, with peak immunoreactivity at ZT6 (0.542 ± 0.025) and lowest immunoreactivity at ZT18 (0.361 ± 0.029).

### 2.3. The Effects of GA and Light on GABA_A_R Subunit Expression in the SCN at Times of Maximal Behavioural Phase Shifts (CT9–CT13)

**Figure 4:**
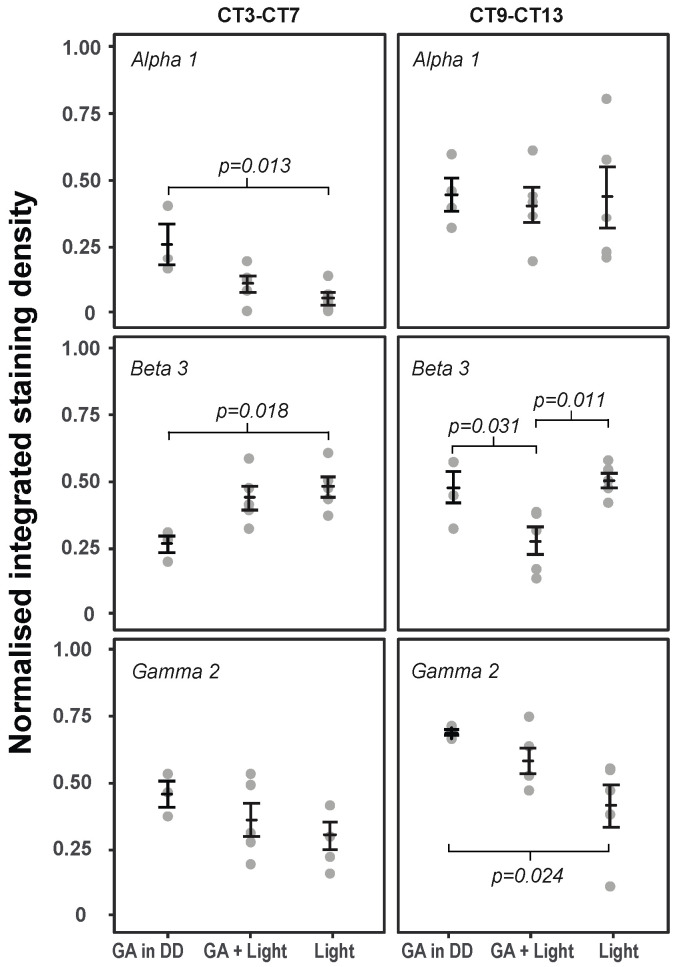
Quantification of GABA_A_R α1, β3, and γ2 subunit immunoreactivity in the mouse SCN in response to GA and light treatments. The data are graphed as individual means ± SEM. (significant differences (Tukey’s HSD (*p* < 0.05)) are shown).

The effect of GA and light on GABA_A_R subunit expression in the SCN was analysed at two different circadian times chosen to correspond with times of minimal (CT3–CT7) and maximal (CT9–CT13) behavioural phase shifting (Figure 4, Table 2, Table 3, (Supplementary Table S2, Supplementary Figure S2)).

At CT9–CT13, where administration of GA induces large behavioural phase delays, the GABAAR γ2 subunit displayed increased immunoreactivity in both the GA in DD and GA + Light treatment groups compared to light alone (and the expression of the γ2 subu-nit in all treatments at CT3–CT7) (Figure 4). Animals receiving GA in DD exhibited the highest γ2 subunit expression in the SCN, which was significantly different (*p* < 0.05) from the light treatment group, with a mean integrated density of 0.698 (± 0.009). The level of γ2 subunit expression in the light group was comparable to expression at ZT12 of 0.423 (± 0.083).

The level of β3 subunit immunoreactivity detected in SCN after GA + Light treatment at CT9–CT13 was significantly lower when compared to expression in the GA in DD and light treatment groups, respectively (*p* < 0.05).

There were no discernable changes in α1 subunit expression in the SCN between any of the treatment groups at CT9–CT13. For all three treatment groups, α1 subunit immuno-reactivity at CT9–CT13 was similar to expression at ZT12 under LD conditions.

### 2.4. The Effects of GA and Light on GABA_A_R Subunit Expression in the SCN at Times of Minimal Behavioural Phase Shifts (CT3–CT7)

At times of minimal behavioural phase shifts, the level of α1 subunit immunoreactivity was significantly increased in the GA in DD treatment group compared to light alone (Figure 4, Table 4). However, when compared to expression under LD 12:12 at ZT6, α1 subunit expression in the SCN was decreased across all treatment groups.

At CT3–CT7, the β3 subunit showed an opposing trend in expression to the α1 subunit, with immunoreactivity significantly increased in the light group compared to GA in DD.

There was no significant difference in γ2 subunit expression in the SCN observed between treatment groups at CT3–CT7.

## 3. Discussion

If the mammalian circadian system was shown to respond in a similar manner to GA and light as honey bees, our previous demonstration in bees that the phase shifting effects of isoflurane can be ameliorated by the concurrent administration of light [7] would provide, at least theoretically, a means to reduce GA-induced phase shifting in mammals. Initial data on the effects of GA on mice in free-running conditions (DD) indicated that, unlike bees, the times of GA-induced phase shifts were not in antiphase to those of light, but occurred at similar circadian times (both GA and light causing delays between CT7 and CT18) [9,10]. The phase response curve presented here for the combined effects of isoflurane (2% for 4 h) and light on behavioural rhythms in mice further these findings. They indicate that the timing of phase delays with GA, light, and GA + Light are all aligned. Thus, concurrent exposure of animals to light and isoflurane will not reduce GA-induced shifts which persist between CT8 and CT18 (Figure 1).

GA-induced behavioural phase shifts not only persist in the presence of a light pulse, the magnitude of these shifts are similar to those induced following isoflurane administration under free-running conditions. If anything, shifts are moderately larger in magnitude than those observed in DD, with a maximum phase delay of −1.05 h (compared to −0.77 h in DD) [9]. The GA + Light PRC appears to sit in-between the PRCs for light alone and GA alone.

Historical work by Colwell et al. (1993) in golden hamsters found that administration of certain GAs (including the inhalational agent halothane) prior to a light pulse eliminated the phase shifting effects of light on behavioural rhythms [27]. The inference of this finding was that general anaesthetics (known to work in part via GABA) prevent light-induced phase shifting. While our behavioural work does suggest an interaction between light and GA (as phase delays in the GA + Light treatment groups are reduced with respect to those in light alone) it clearly indicates that light-induced phase shifting persists in the presence of clinical doses of isoflurane.

Previous work to suggest that GABA_A_ receptors may mediate the effects of GA on the clock have relied on the approach of blocking GABA and examining the resulting effect on phase shifting light pulses. Our approach to the investigation of the putative role of GABA in GA-induced phase shifting was to investigate GABA_A_R expression in the SCN following behavioural phase shifts induced by GA, light or GA + Light.

Surprisingly, given the proposed role of GABA in the entrainment and coupling of the mammalian clock, there is a paucity of information on the presence and temporal pattern of expression of different GABA_A_R subunits in the mouse SCN. Here, we have confirmed that three different GABA_A_R subunits (α1, β3, and γ2) are expressed in the SCN and that they all show daily variations in their expression levels (with α1 peaking at ZT6 and β3, and γ2 peaking between ZT18 and ZT0). These findings are similar those of Walton et al. (2017) who found GABA_A_R γ2 protein immunoreactivity in the SCN of Syrian hamsters to peak during the night (around ZT13) [28]. In DD these authors showed an increased expression during the “subjective day” (at CT6) which they interpret as evidence of light inhibition of γ2 subunit expression.

The confirmation of the expression of α1, β3, and γ2 GABA_A_R subunits in the SCN of C57 mice enabled us to determine expression changes due to GA, light and GA + Light in the SCN, and to examine whether changes in expression may correlate with the behavioural phase shifts caused by these agents. The circadian times at which GA, light and GA+ light cause maximal phase delays (CT9-CT13) was, of course, of primary interest as these are times when the phase of the clock is affected. If GABA is in fact involved in this shifting one might expect to see changes in the receptors expressed in the SCN. The expression of γ2 in the SCN following GA (in DD) is significantly higher than in the light treatment group (and, while not significant the light and GA treatment group falls between these two) (Figure 3). This parallels the behavioural effects of GA, GA + Light and light (Figure 1). Our interpretation of these data is that either GA is resulting in an increase in γ2 expression in the SCN or that light is suppressing γ2 expression but that the light suppression of γ2 is partially inhibited by the presence of GA. The expression of α1 shows no significant changes due to treatment at CT9-CT13, and the expression of β3 shows a significant reduction in expression in the GA + Light group compared to the other two treatments.

At circadian times during which no, or minimal, behavioural phase shifts result from the administration of light or GA, or the two agents together (i.e., CT3-CT7), there are some significant changes in GABA_A_R subunit expression in the SCN, with a reduction in α1 expression in the light group (compared to GA in DD), and an increase in β3 expression in light group (compared to GA in DD). Given behavioural phase shifts are not observed at these CTs any link between changes of subunit expression at these circadian times to a shift of the clock would seem unlikely.

Taken together, these expression data provide preliminary evidence to suggest that the GABA_A_R γ2 subunit, and its associated receptor configurations, could potentially play a part in mediating GA-induced behavioural phase shifts in mice.

Certainly the expression of the γ2 subunit is highest in the GA treatment group at circadian times which induce behavioural shifts. The apparent reduction in expression when GA and light are administered together, and the significant reduction when light alone is given, does not correspond with the magnitude of behavioural phase shifts in the three groups (i.e., Light alone induces the largest phase delays). While these results may appear difficult to reconcile, an interpretation of why light and GA could affect γ2 subunit expression differently may be provided by the results of Walton et al. (2017) [28]. In their paper, they propose that light phase shifts at different CTs are mediated by different receptor configurations. If this observation were extended to the effects of different *zeitgeber,* then these apparently contradictory results may be explicable.

In conclusion, concurrent administration of GA and light does elicit behavioural phase shifts in a time-dependent manner in mice. The effect of light on the clock are not blocked by the administration of general anaesthesia in mice. However, if light is to be used to counter the phase shifting effects of GA in mice then it cannot be administered concurrently (as in bees). For example, in order to counteract the phase delaying effects of GA at CT14 light “treatment” would need to be timed to occur when it has a maximal phase advance.

The GA-induced shifts shown here (and those in our previous paper [9]) may be mediated, at least in part, through the activation of different GABA_A_R subunits or configurations within the SCN. Our demonstration here that three different GABA_A_R subunits are expressed in the mouse SCN and show strong temporal changes over the course of the day, highlights the complex and dynamic role GABA is likely to play in the central clock. A systematic investigation of the expression of all of the different GABA_A_R subunits in the SCN would seem an important next step in determining the role of GABA in the regulation and entrainment of the clock (and GAs effect on the clock). It is important to note that the expression changes shown here do not imply a functional role for GABA_A_R in the GA-induced phase shifting of the circadian clock. Further functional studies using GABA and non-GABA acting anaesthetics, and GABA agonists and antagonists will be necessary to fully elucidate GABA’s role in GA-induced phase shifting.

## 4. Materials and Methods

All procedures were approved and performed in accordance with the regulations by the University of Auckland Animal Ethics Committee (approval #002138). All experiments were performed on eight- to twelve-week-old male C57BL/6 mice supplied by the Vernon Jansen Unit, University of Auckland.

### 4.1. Behavioural Experiments

Animals were housed individually in standard transparent cages (Coulbourn Instruments, (Allentown, MA, USA) fitted with a rodent running wheel attached to an infrared microswitch (Actimetrics, Wilmette, IL, USA) and had access to food and water ad libitum. Running wheel activity was recorded for circadian analysis using the Clocklab system (Actimetrics, Wilmette, IL, USA). Cages were maintained in ventilated, sound attenuated, temperature controlled environmental chambers for the duration of the experiments at 23 °C +/− 1 °C.

Locomotor activity rhythms of individual animals were recorded for a period of 40 days. Each animal received either a 4 h light exposure (control) or a 4 h GA (with isoflurane) during which they received the same light exposure but only while anaesthetized.

This was achieved using the following protocol: after 10 days of light cycles (LD 12:12) animals were transferred into constant darkness (DD) for 14 days prior to their treatment. Their activity was then monitored for a further 14 days prior to the end of the experiment and euthanasia. As the mouse circadian clock is highly light sensitive, during DD all procedures (including GA) were conducted in complete darkness using night vision goggles.

In all cases, GA with isoflurane was conducted as previously described in [9]. Anaesthetic induction with 5% isoflurane (4 L/min) was achieved within 10–30 s, after which the anaesthetic concentration was reduced and maintained at 1.5–2.0% (4 L/min) for the duration of treatment. Anaesthesia was deemed to occur when animals showed a loss of righting reflex and no response to tail pinch. Spontaneous respiratory rate was monitored (visually) every five minutes and maintained above 30 breaths/min at all times. In the event that respiratory rate dropped below 30, animals were placed in a second oxygen chamber (without isoflurane) until respiratory rate had recovered, at which point they were returned to the anaesthetic chamber. Using this protocol mortality rate from anaesthesia was zero. After three and a half hours of GA the vaporizer was switched off, and animals were partially recovered in the chamber and then returned to their individual cages prior to emergence. Emergence time (as determined by return of righting reflex) varied between individuals but typically took 20–25 min.

### 4.2. Behavioural Analysis

Circadian times of treatment were calculated using CT12 (manufacturer, city, state abbreviation if USA or Canada, country) (onset of activity using the Clocklab function using the 20th percentile of activity levels (https://www.harvardapparatus.com/media/manuals/Product%20Manuals/ACT-500%20ClockLab%20Analysis%20Manual.pdf, accessed on 14 September 2021)) as a phase reference point, and the midpoint of the GA as the time at which GA occurred. Free-running periods of behavioural rhythms were calculated prior to and after GA using the ꭓ2 periodogram analysis function in Clocklab. Shifts in the phase of behavioural rhythms were calculated (using onset of activity as the phase reference point and a least squares fit through the onsets) on the seven days prior to and following the administration of GA.

### 4.3. Immunohistochemistry Experiments

To assess GABA_A_R expression in the SCN at different times of the day (results described in Section 2.2), animals were maintained in LD 12:12 for a period of seven days and entrainment to the LD cycle for a minimum of three days was confirmed. Brains were collected at *zeitgeber* time (ZT)0, ZT6, ZT12, and ZT18, with collections at ZT12 and ZT18 conducted in darkness.

To determine the effect of GA, GA + Light and Light on GABA_A_R expression in the SCN (results described in Section 2.3 and Section 2.4) animals were maintained on LD cycles for seven days (as above) and were then transferred into DD for a period of two weeks prior to brains collection at two different circadian time bins CT3–CT7 and CT9–CT13.

In order to collect the brains for staining, animals were deeply anaesthetized with an overdose of 75 mg/kg ketamine and 1 mg/kg medetomidine and transcardially perfused with 25 mL of ice-cold 4% paraformaldehyde (PFA) in phosphate buffer (pH 7.6). Brains were removed immediately, postfixed in 4% PFA for 2 h at room temperature (RT) and then placed in 30% sucrose in Tris-buffered saline (TBS, pH 7.6) solution overnight at 4 °C. Four sets of serial coronal brain sections (30-µm thickness) were cut using a freezing microtome and stored in antifreeze solution at −20 °C.

### 4.4. Immunohistochemistry

Free-floating single-label fluorescence immunohistochemistry was performed to detect GABA_A_R α1, β3, and γ2 subunits within the mouse SCN as previously described in [29]. Specificity of the primary antibodies has been tested using Western blotting and reported previously [30,31,32]. Tissue sections were first incubated in blocking solution containing 1% donkey serum in 0.05 M TBS/0.3% *v/v* Triton X-100/0.25% *w/v* BSA (TTB) for 1 h at RT. Sections were then washed in TBS for 3 × 10 min prior to incubation with the appropriate primary antibody (α1-rabbit anti-alpha 1, Alomone Laboratories, AGA-001, Jerusalem, Israel (1:1000); β3-mouse anti-beta 3, Novus, NBP-1-47613, Toronto, Canada (1:500); γ2-rabbit anti-gamma 2, Synaptic Systems, 224-003, Goettingen, Germany (1:1000)) for 48 h at 4 °C.

Sections were then incubated with the appropriate secondary antibody (α1: -donkey anti-rabbit Alexa Fluor 647, ThermoFisher Scientific, A-31573, Waltham, MA, USA (1:500); β3: - donkey anti-mouse, Alexa Fluor 488, ThermoFisher Scientific A-21202 (1:500) γ2: - donkey anti-rabbit Alexa Fluor 647, ThermoFisher Scientific, A-31573, (1:500)) for 1 h at RT. Cell nuclei were counterstained with Hoechst 33,342 (Invitrogen, Carlsbad, CA, USA (1:10,000)) for 30 min to distinguish the boundary of the SCN. Sections were mounted onto slides, air dried overnight at RT, and then coverslipped with Mowiol mounting medium. Sections incubated without primary antibody were used as negative controls. Omission of the primary antibody resulted in completed absence of immunoreactivity.

### 4.5. Imaging and Analysis

For quantification, imaging was conducted using a Zeiss LSM 710 inverted confocal laser-scanning microscope (Carl Zeiss, Jena, Germany) with a 20x objective (Plan-Apochromat 20x/0.8 NA, Carl Zeiss, GmbH, Germany). The boundary of each nucleus of the SCN was determined using Hoechst staining. Receptor density measurements were undertaken using ImageJ (version 1, 50I, National Institutes of Health, Bethesda, MD, USA). After background subtraction (correcting for uneven illuminated background) and gray scale threshold determination (correcting for saturated pixels), the GABA_A_R subunit density measurements were performed on each unilateral SCN and these values were averaged (across the left and right nuclei) to produce a single density value. These measurements were expressed with respect to the size of the SCN to obtain an integrated SCN density before averaging across tissue sections (with a minimum of three tissue sections measured per animal) and within treatment groups. Integrated SCN density was normalized to the range of density values in a given immunohistochemistry run for each GABA_A_R subunit. The experimenter was blinded to the experimental groupings to eliminate any bias during experiment, including image acquisition and analysis.

### 4.6. Statistical Analysis

Differences in staining intensity between the different times of the day and between different treatment groups were each tested using a ANOVA with a post hoc test (Tukey’s HSD). The ANOVA assumption of homogeneous variances was checked using Levene’s test. No evidence of heteroscedasticity was found in each case. Differences were deemed significant at the *p* < 0.05 level.

## Figures and Tables

**Table 1:** Summary statistics of circadian times (CT) and phase shifts for behavioural phase shifting experiments with Light (400 lux, 4 h) and GA (2% isoflurane) + Light (400 lux). Shifts from GA in DD (2%, 6 h) are included in grey for the sake of comparison.

	Light		GA+Light		GA in DD (Ludin et al., 2021)
CT (range)	*n*	Shift (SE) (h)	*n*	Shift (SE) (h)	Shift (SE) (h)
2 (0.4–3.8)	11	0.30 (0.13)	7	0.08 (0.16)	0.17 (0.14)
6 (4.1–7.9)	11	0.33 (0.16)	10	−0.16 (0.21)	− 0.11 (0.38)
10 (8.0-–2.0)	8	0.53 (0.17)	9	−0.82 (0.52)	− 0.61 (0.31)
14 (14.2–15.9)	6	−3.22 (0.29)	6	−1.87 (0.26)	− 0.77 (0.18)
18 (16–19.6)	15	−3.27 (0.21)	14	−0.45 (0.20)	− 0.25 (0.11)
22 (20.2–24)	7	−1.08 (0.69)	14	0.25 (0.12)	0.23 (0.14)

**Table 2:** Summary statistics of GABA_A_R subunit expression under LD conditions. Evidence of differences between the different ZTs was tested using ANOVA. Different ZTs that share the same superscript indicate the difference between them is statistically significant (Tukey’s HSD *p* < 0.05) e.g., ^1^ denotes a difference between ZT6 and ZT12 for alpha 1.

	ZT0		ZT6		ZT12		ZT18	
	*n*	Mean (SE)	*n*	Mean (SE)	*n*	Mean (SE)	*n*	Mean (SE)
Alpha 1	5	0.462 (0.015)	6	0.542 (0.025) ^1,2^	5	0.411 (0.042) ^1^	6	0.361 (0.029) ^2^
Beta 3	2	0.728 (0.057) ^3^	4	0.400 (0.066) ^3^	4	0.456 (0.057)	5	0.568 (0.065)
Gamma 2	3	0.633 (0.019)	4	0.426 (0.046)	6	0.412 (0.066) ^4^	6	0.609 (0.025) ^4^

**Table 3:** Summary statistics of GABA_A_R subunit expression during late circadian times at which phase shifts due to light and GA are maximal (CT9–CT13). Evidence of differences between treatment groups was tested using ANOVA. Different treatments that share the same superscript number indicate the difference between them is statistically significant (Tukey’s HSD *p* < 0.05).

Treatment	Light		GA + Light		GA in DD	
	*n*	Mean (SE)	*n*	Mean (SE)	*n*	Mean (SE)
Alpha 1	5	0.440 (0.114)	5	0.409 (0.067)	4	0.449 (0.059)
Beta 3	5	0.526 (0.027) ^1^	5	0.284 (0.052) ^1,2^	4	0.486 (0.061) ^2^
Gamma 2	5	0.423 (0.083) ^3^	5	0.592 (0.051)	4	0.698 (0.009) ^3^

**Table 4:** Summary statistics of GABA_A_R subunit expression during early circadian times at which phase shifts due to light and GA are negligible (CT3–CT7). Evidence of differences between treatment groups was tested by ANOVA. Different treatments that share the same superscript number indicate the difference between them is statistically significant (Tukey’s HSD *p* < 0.05).

Treatment	Light		GA + Light		GA in DD	
	*n*	Mean (SE)	*n*	Mean (SE)	*n*	Mean (SE)
Alpha 1	5	0.056 (0.024) ^1^	5	0.113 (0.031)	3	0.261 (0.073) ^1^
Beta 3	5	0.486 (0.039) ^2^	5	0.445 (0.045)	3	0.271 (0.034) ^2^
Gamma 2	5	0.313 (0.051)	5	0.371 (0.065)	3	0.467 (0.046)

## Data Availability

Not applicable.

## Supplementary Materials

The following are available online at https://www.mdpi.com/article/10.3390/clockssleep3030034/s1.
