# Supplementary material for: The Effects of General Anaesthesia and Light on Behavioural Rhythms and GABAA Receptor Subunit Expression in the Mouse SCN
Source: Clocks Sleep. 2021 Sep 17;3(3):482–94. doi: 10.3390/clockssleep3030034 (PMC8482144; doi:10.3390/clockssleep3030034)

Supplementary Materials:

Figure S1: Behavioural actograms of 60 C57BL6/VJU mice exposed to 4 hours of GA (2% isoflurane) + Light

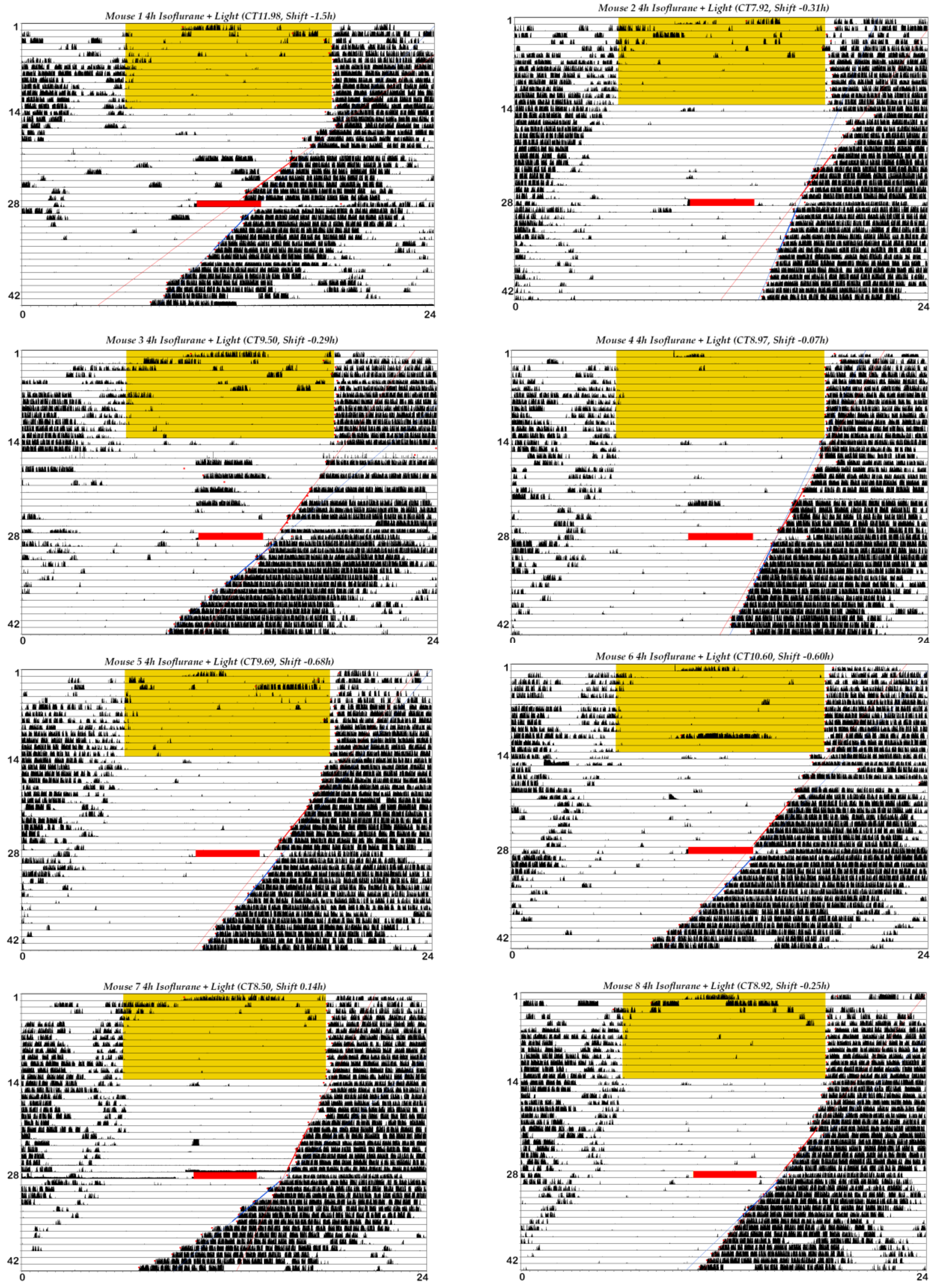

Mouse 9 4h Isoflurane + Light (CT8.53, Shift 0.60h)

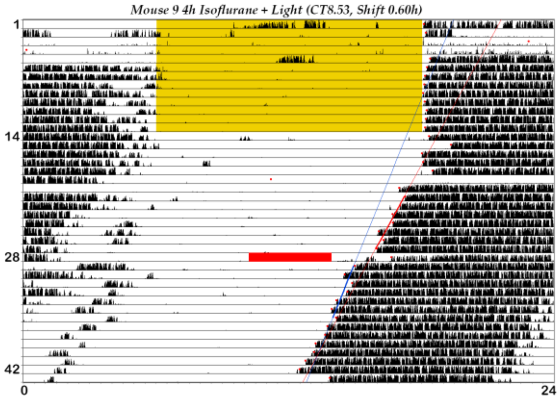

Mouse 10 4h Isoflurane + Light (CT8.02, Shift -4.70h)

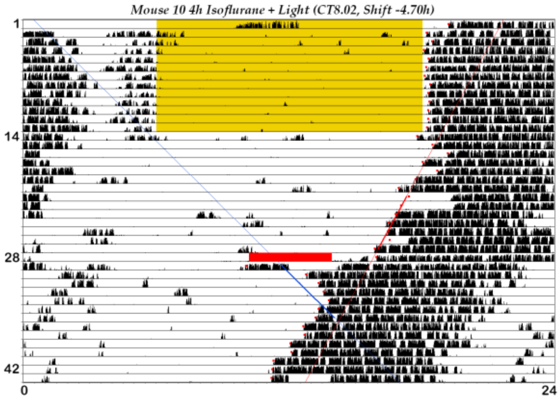

Mouse 11 4h Isoflurane + Light (CT3.34, Shift -0.35h)

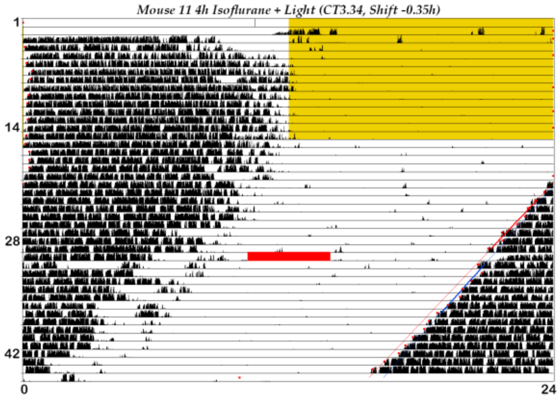

Mouse 12 4h Isoflurane + Light (CT23.91, Shift 0.05h)

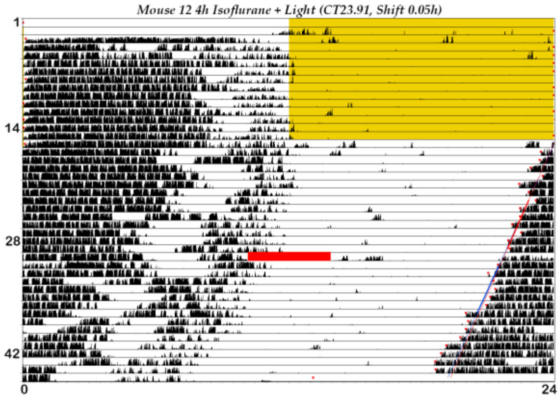

Mouse 13 4h Isoflurane + Light (CT3.03, Shift 0.20h)

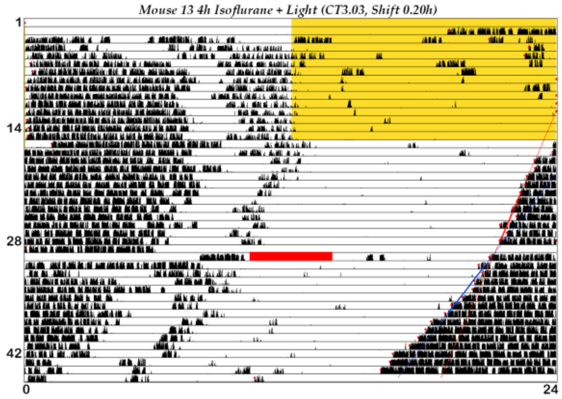

Mouse 14 4h Isoflurane + Light (CT2.59, Shift -0.27h)

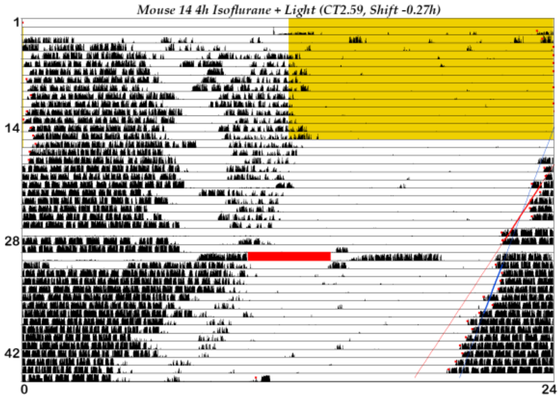

Mouse 15 4h Isoflurane + Light (CT23.75, Shift 0.63h)

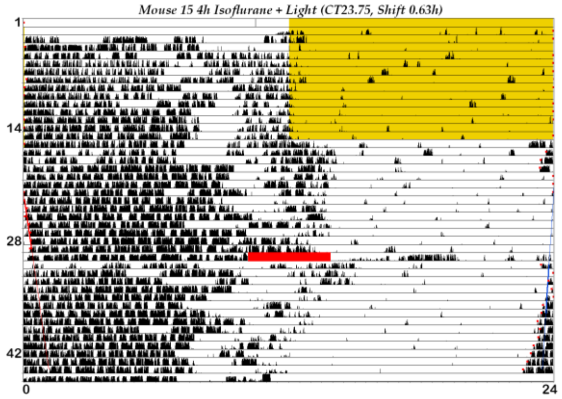

Mouse 16 4h Isoflurane + Light (CT23.40, Shift 0.66h)

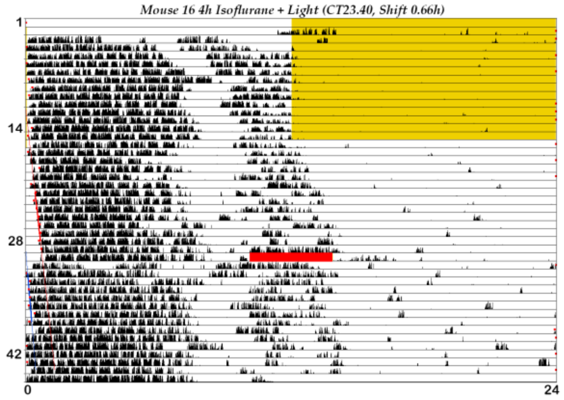

Mouse 17 4h Isoflurane + Light (CT23.84, Shift 0.33h)

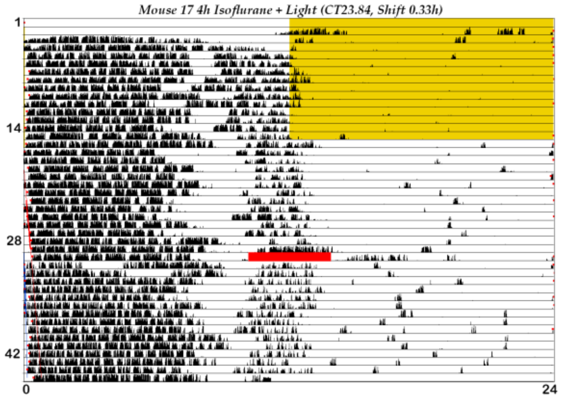

Mouse 18 4h Isoflurane + Light (CT0.58, Shift 0.48h)

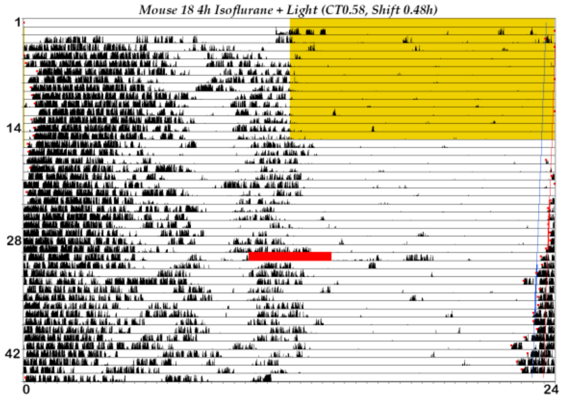

Mouse 19 4h Isoflurane + Light (CT2.40, Shift -0.27h)

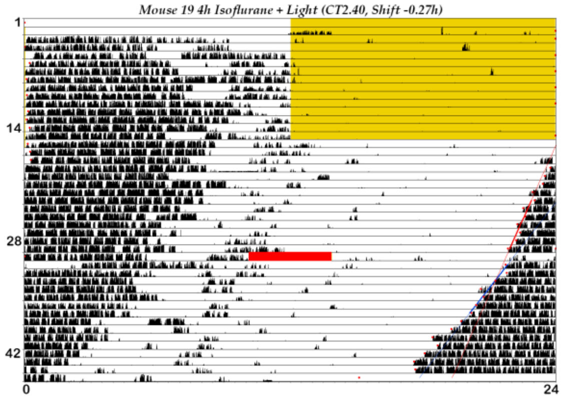

Mouse 20 4h Isoflurane + Light (CT1.54, Shift 0.94h)

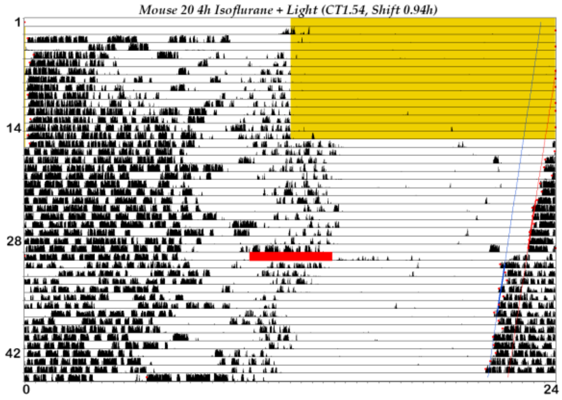

Mouse 21 4h Isoflurane + Light (CT4.67, Shift -1.75h)

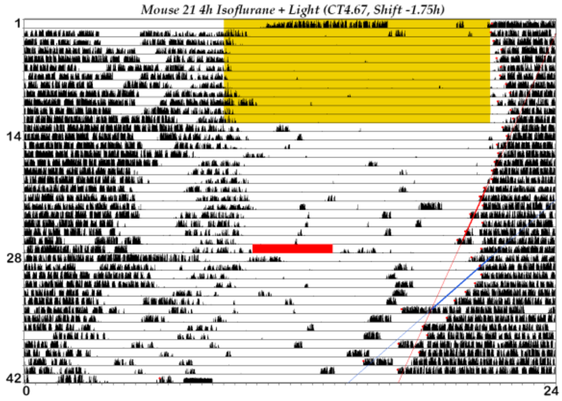

Mouse 22 4h Isoflurane + Light (CT6.81, Shift -0.33h)

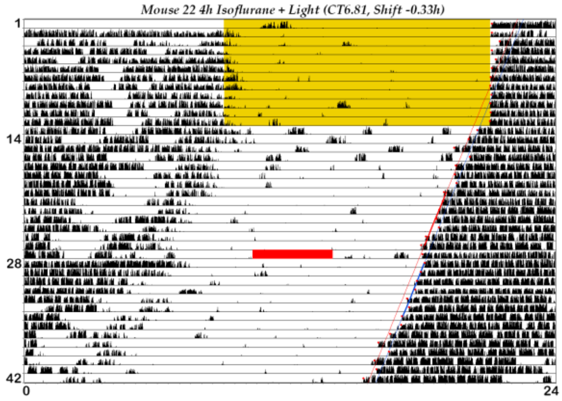

Mouse 23 4h Isoflurane + Light (CT6.38, Shift 0.15h)

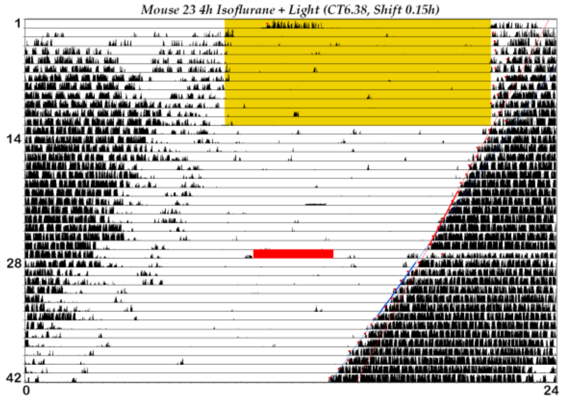

Mouse 24 4h Isoflurane + Light (CT7.82, Shift 0.12h)

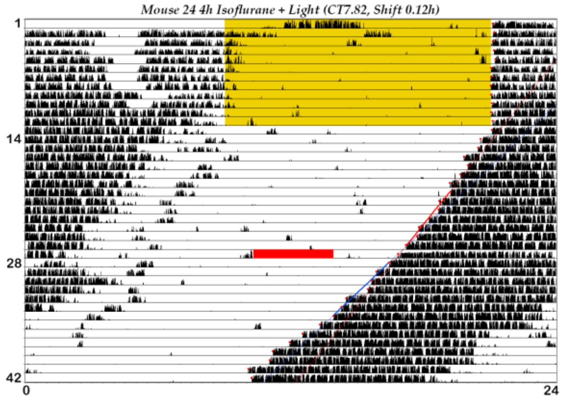

Mouse 25 4h Isoflurane + Light (CT5.24, Shift 0.63h)

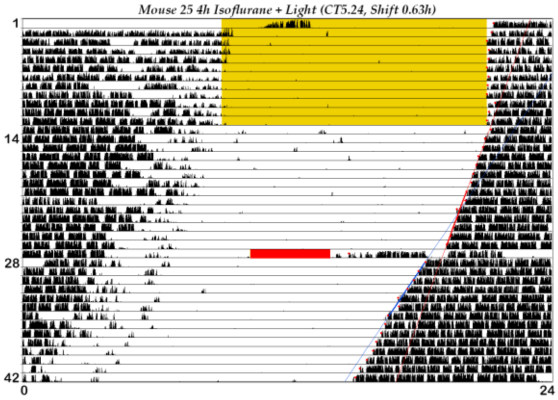

Mouse 26 4h Isoflurane + Light (CT3.22, Shift -0.07h)

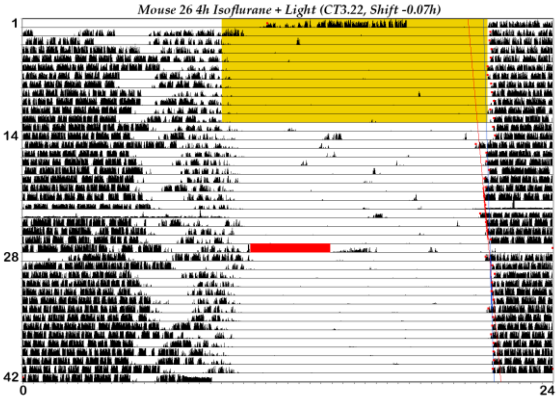

Mouse 27 4h Isoflurane + Light (CT5.17, Shift -0.39h)

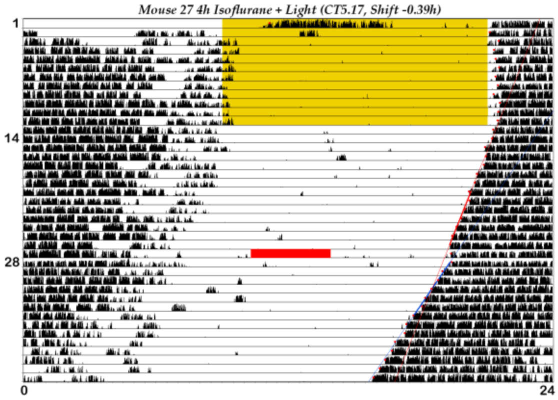

Mouse 28 4h Isoflurane + Light (CT4.05, Shift 0.54h)

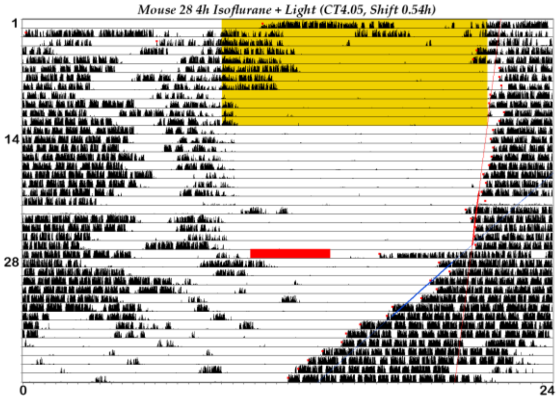

Mouse 29 4h Isoflurane + Light (CT6.69, Shift 0.01h)

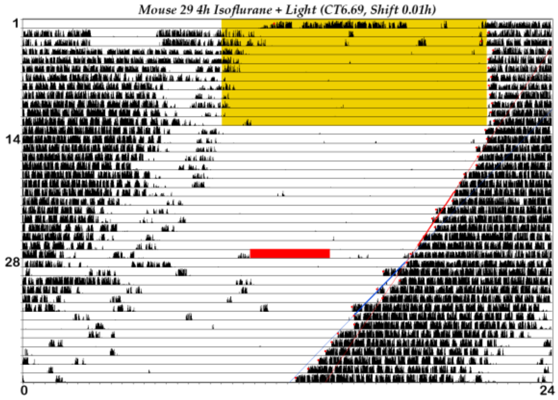

Mouse 30 4h Isoflurane + Light (CT6.70, Shift -0.24h)

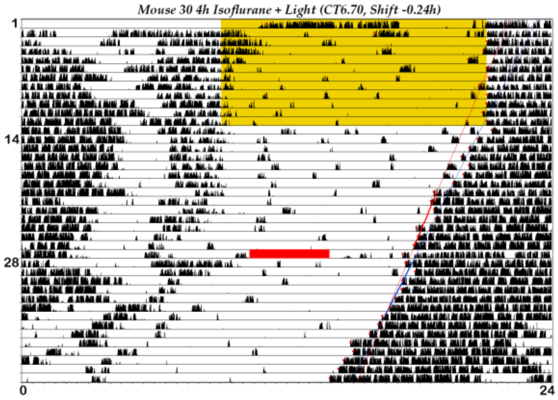

Mouse 31 4h Isoflurane + Light (CT23.97, Shift 0.32h)

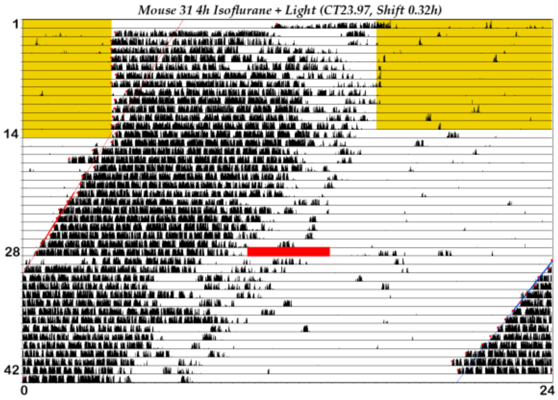

Mouse 32 4h Isoflurane + Light (CT21.60, Shift 0.21h)

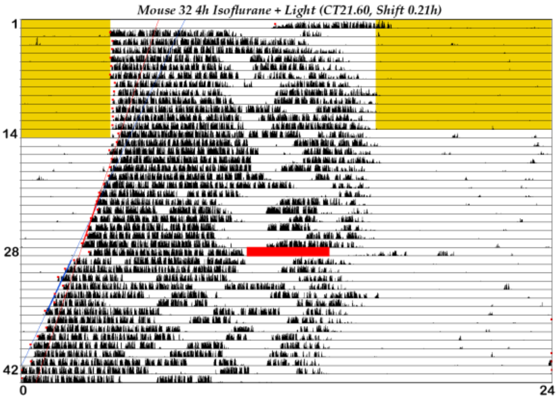

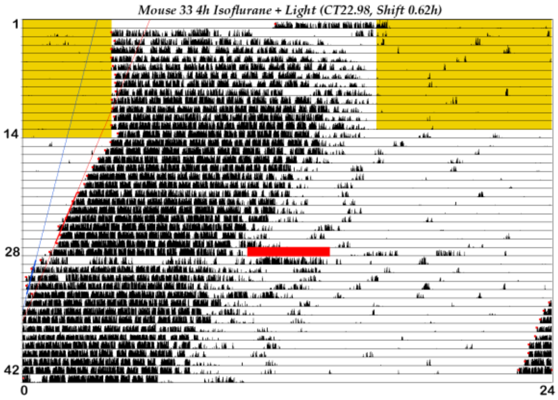

Mouse 33 4h Isoflurane + Light (CT22.98, Shift 0.62h)

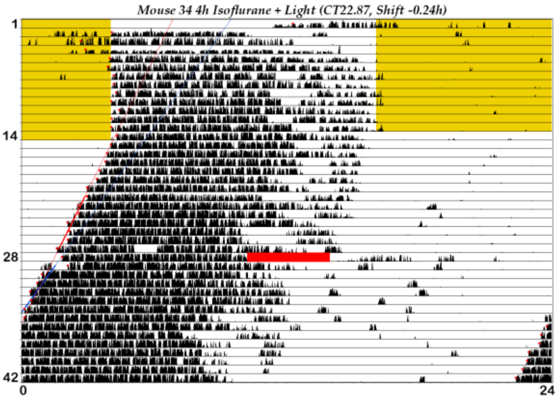

Mouse 34 4h Isoflurane + Light (CT22.87, Shift -0.24h)

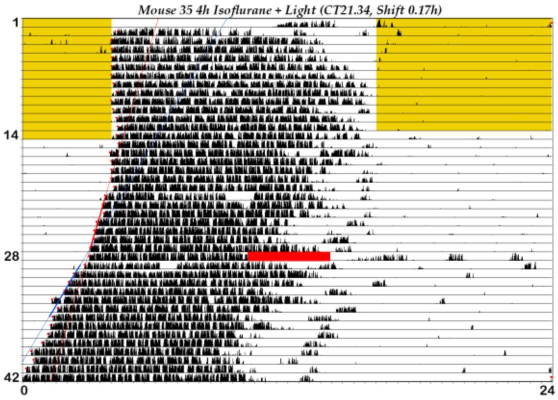

Mouse 35 4h Isoflurane + Light (CT21.34, Shift 0.17h)

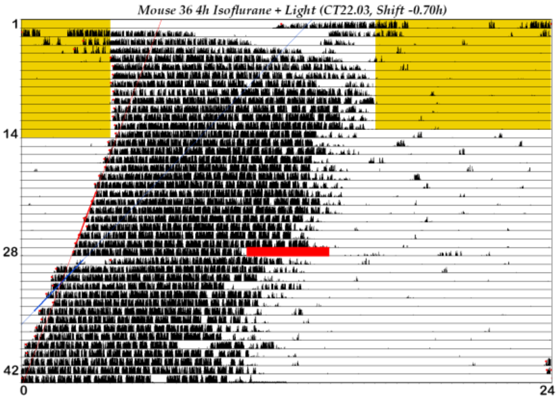

Mouse 36 4h Isoflurane + Light (CT22.03, Shift -0.70h)

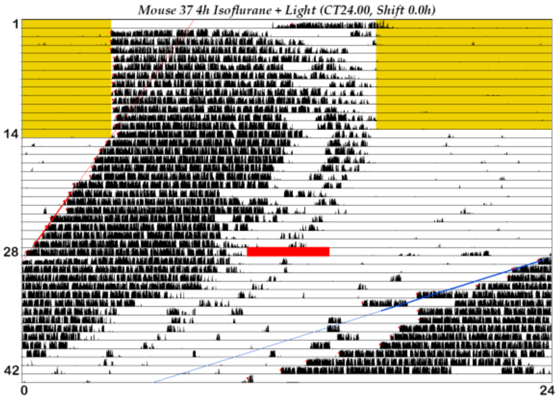

Mouse 37 4h Isoflurane + Light (CT24.00, Shift 0.0h)

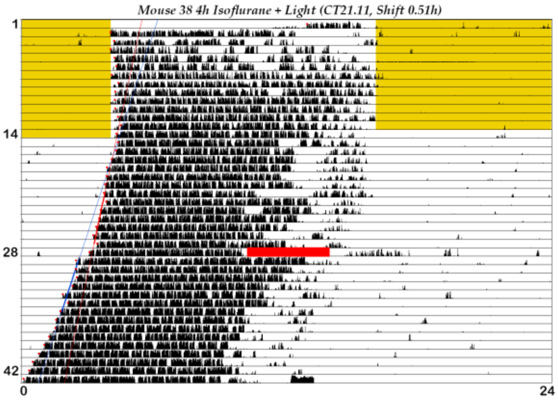

Mouse 38 4h Isoflurane + Light (CT21.11, Shift 0.51h)

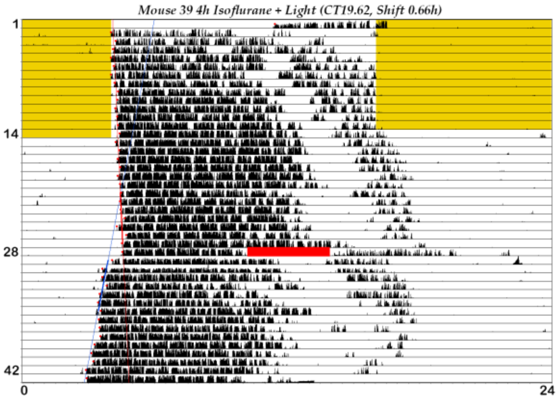

Mouse 39 4h Isoflurane + Light (CT19.62, Shift 0.66h)

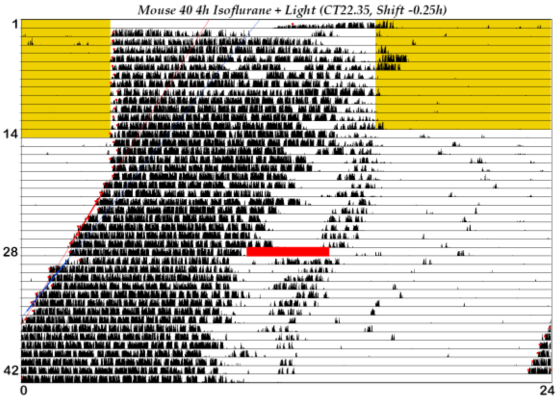

Mouse 40 4h Isoflurane + Light (CT22.35, Shift -0.25h)

Mouse 41 4h Isoflurane + Light (CT15.51, Shift -1.18h)

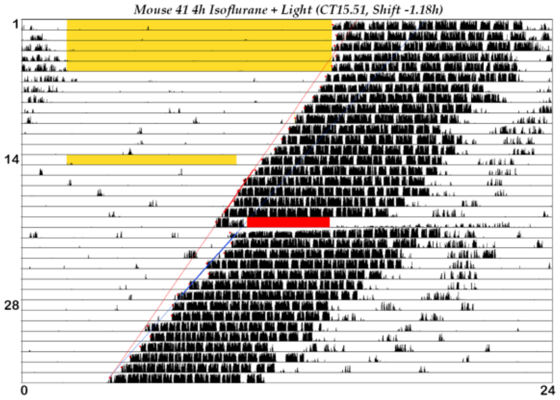

Mouse 42 4h Isoflurane + Light (CT17.15, Shift -0.60h)

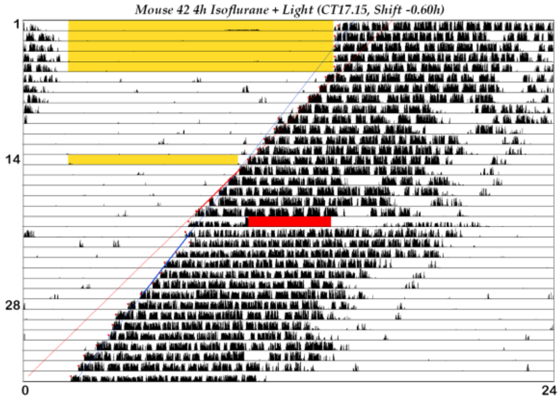

Mouse 43 4h Isoflurane + Light (CT14.17, Shift -2.41h)

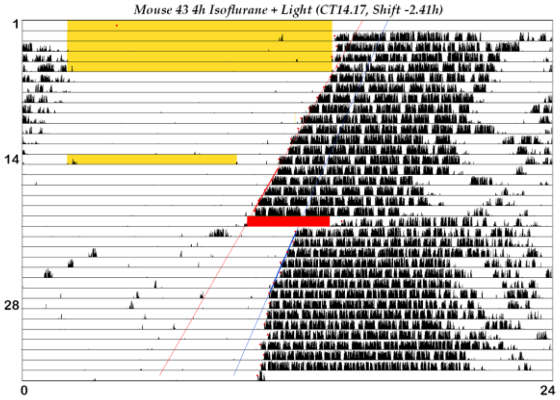

Mouse 44 4h Isoflurane + Light (CT16.68, Shift -0.67h)

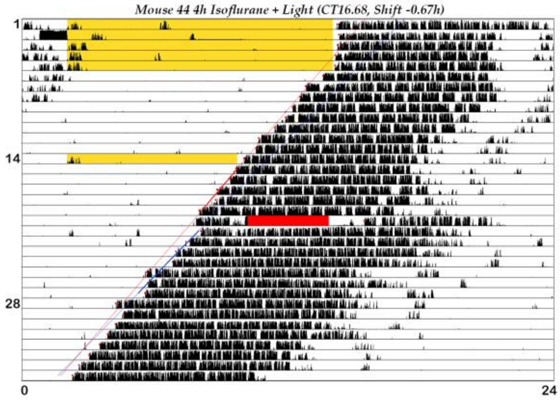

Mouse 45 4h Isoflurane + Light (CT17.20, Shift -1.21h)

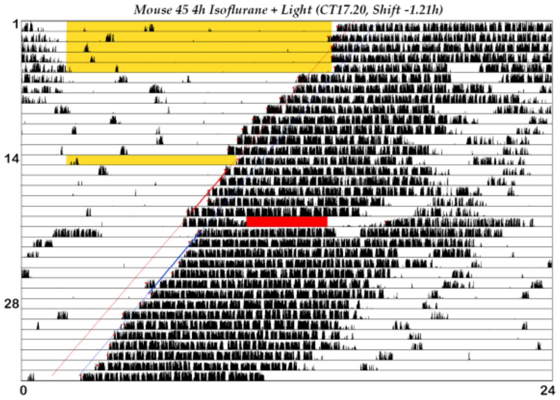

Mouse 46 4h Isoflurane + Light (CT16.89, Shift 0.24h)

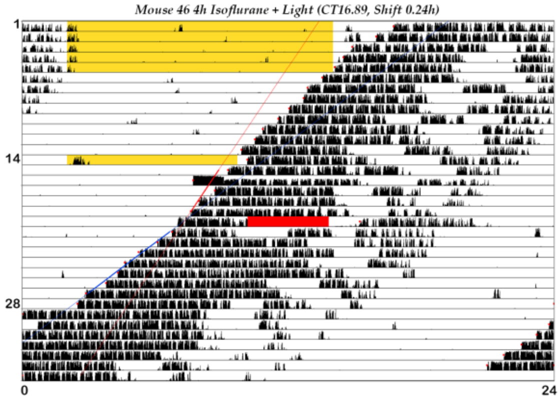

Mouse 47 4h Isoflurane + Light (CT17.66, Shift -1.07h)

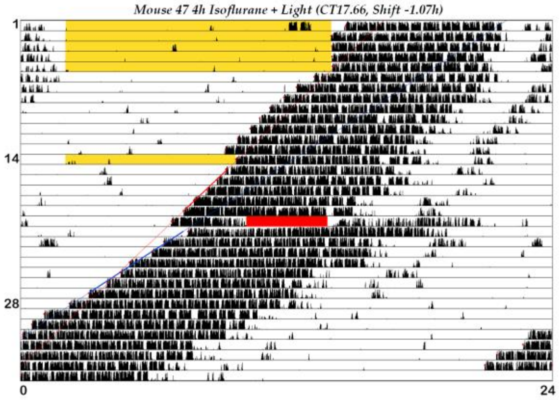

Mouse 48 4h Isoflurane + Light (CT16.97, Shift -1.89h)

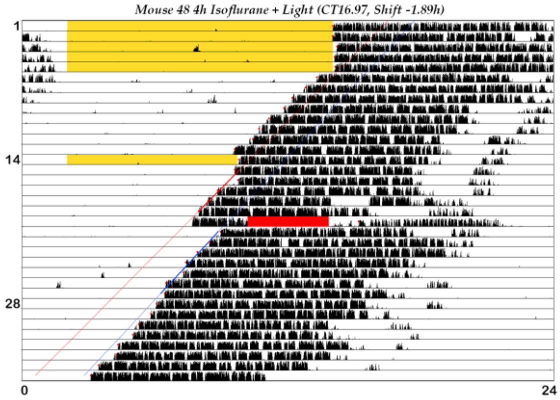

Mouse 49 4h Isoflurane + Light (CT20.83, Shift 0.93h)

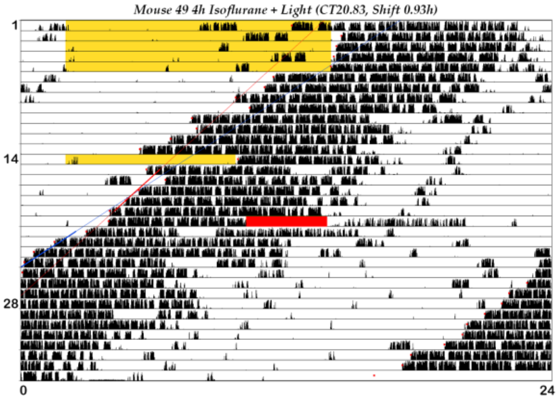

Mouse 50 4h Isoflurane + Light (CT14.36, Shift -2.70h)

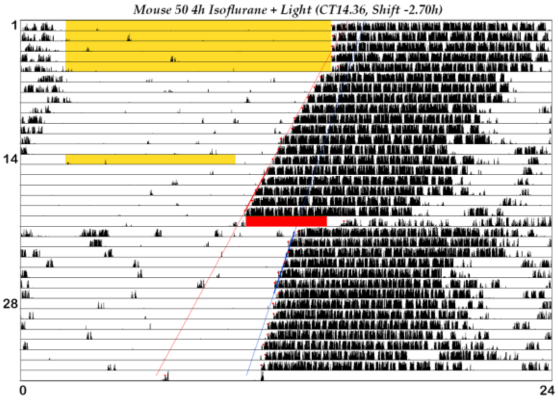

Mouse 51 4h Isoflurane +Light (CT16.78, Shift 0.61h)

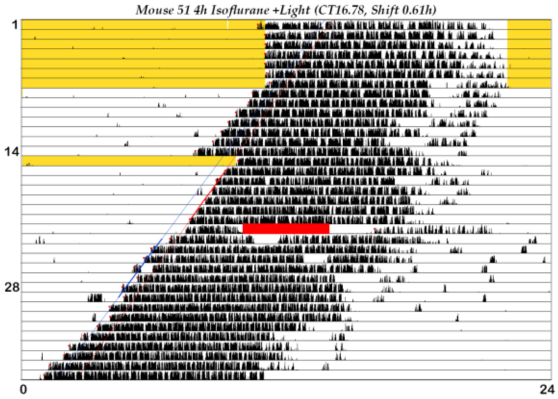

Mouse 52 4h Isoflurane +Light (CT15.79, Shift -2.05h)

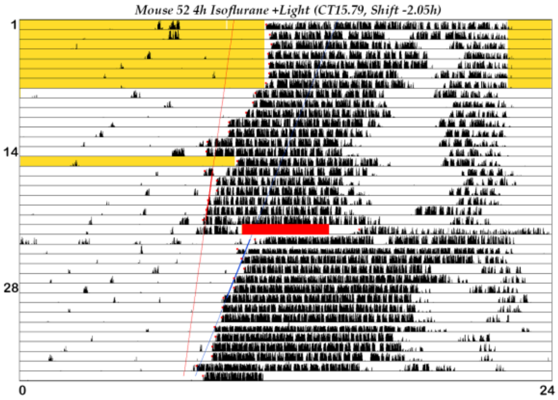

Mouse 53 4h Isoflurane +Light (CT18.20, Shift 0.41h)

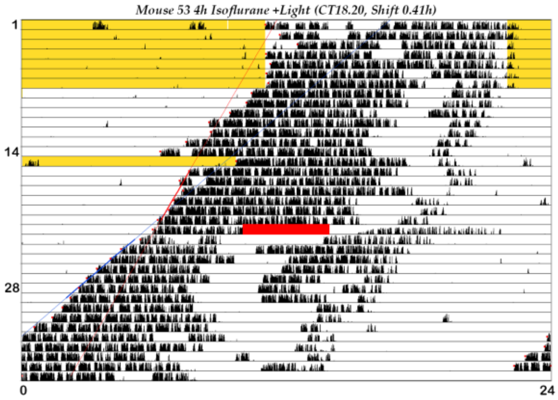

Mouse 54 4h Isoflurane +Light (CT15.27, Shift -1.69h)

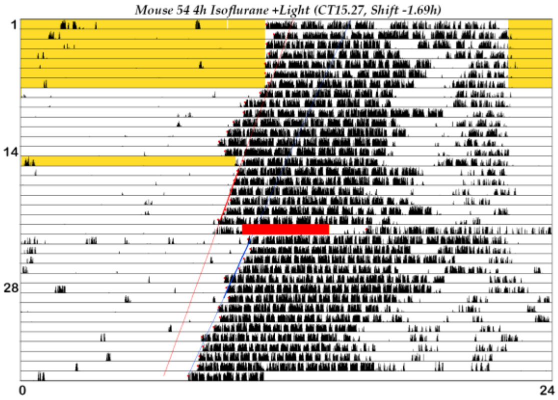

Mouse 55 4h Isoflurane +Light (CT16.84, Shift -0.97h)

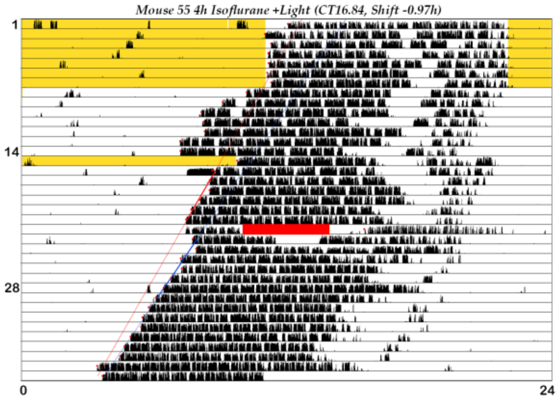

Mouse 56 4h Isoflurane +Light (CT16.50, Shift -0.14h)

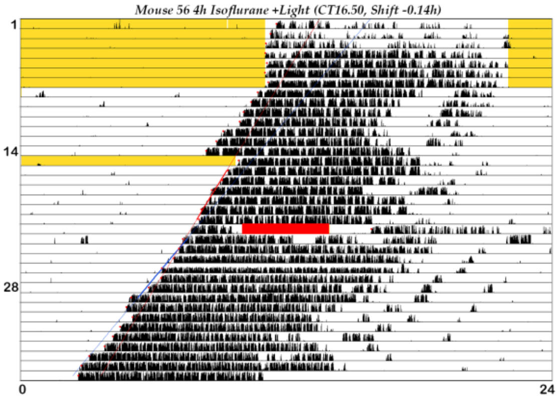

Mouse 57 4h Isoflurane +Light (CT14.97, Shift -1.17h)

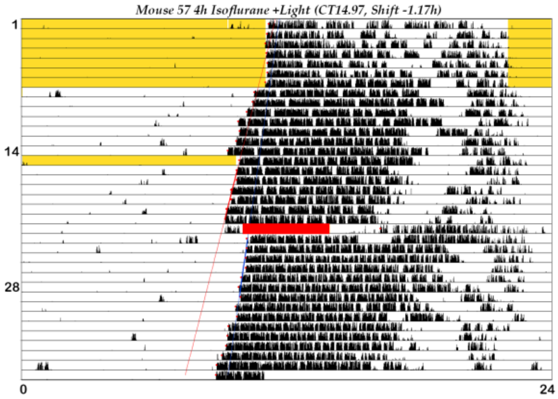

Mouse 58 4h Isoflurane +Light (CT16.64, Shift -0.04h)

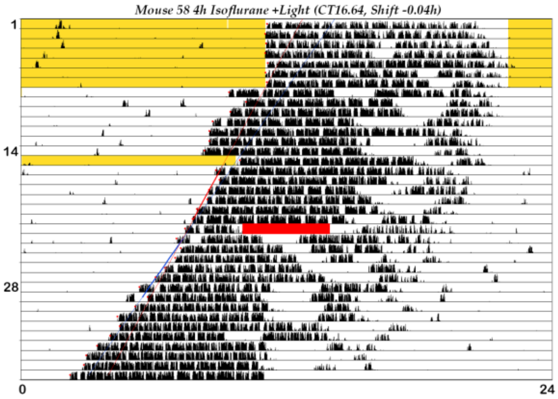

Mouse 59 4h Isoflurane +Light (CT16.87, Shift -0.68h)

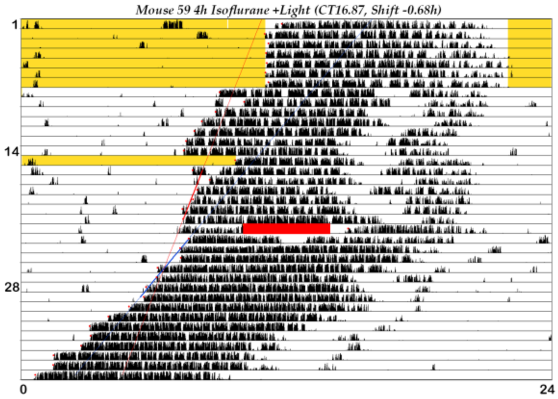

Mouse 60 4h Isoflurane +Light (CT17.02, Shift -1.00h)

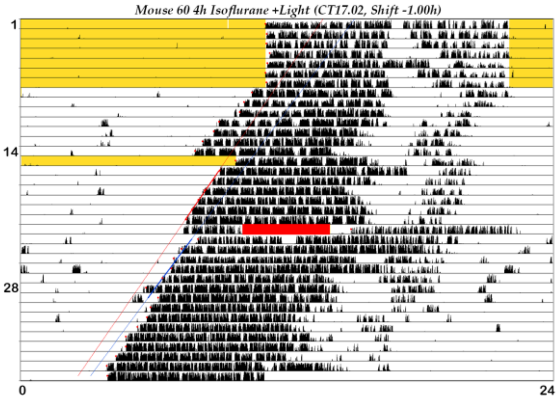

Supplement: Supplementary file 1 [file clockssleep-03-00034-s001.zip › Supplementary Figure S1.pdf]
