# Supplementary material for: The Effects of General Anaesthesia and Light on Behavioural Rhythms and GABAA Receptor Subunit Expression in the Mouse SCN
Source: Clocks Sleep. 2021 Sep 17;3(3):482–94. doi: 10.3390/clockssleep3030034 (PMC8482144; doi:10.3390/clockssleep3030034)

## Supplementary Materials:

**Figure S2: Behavioural actograms of 60 C57BL6/VJU mice exposed to 4 hours of light (400 lux)**

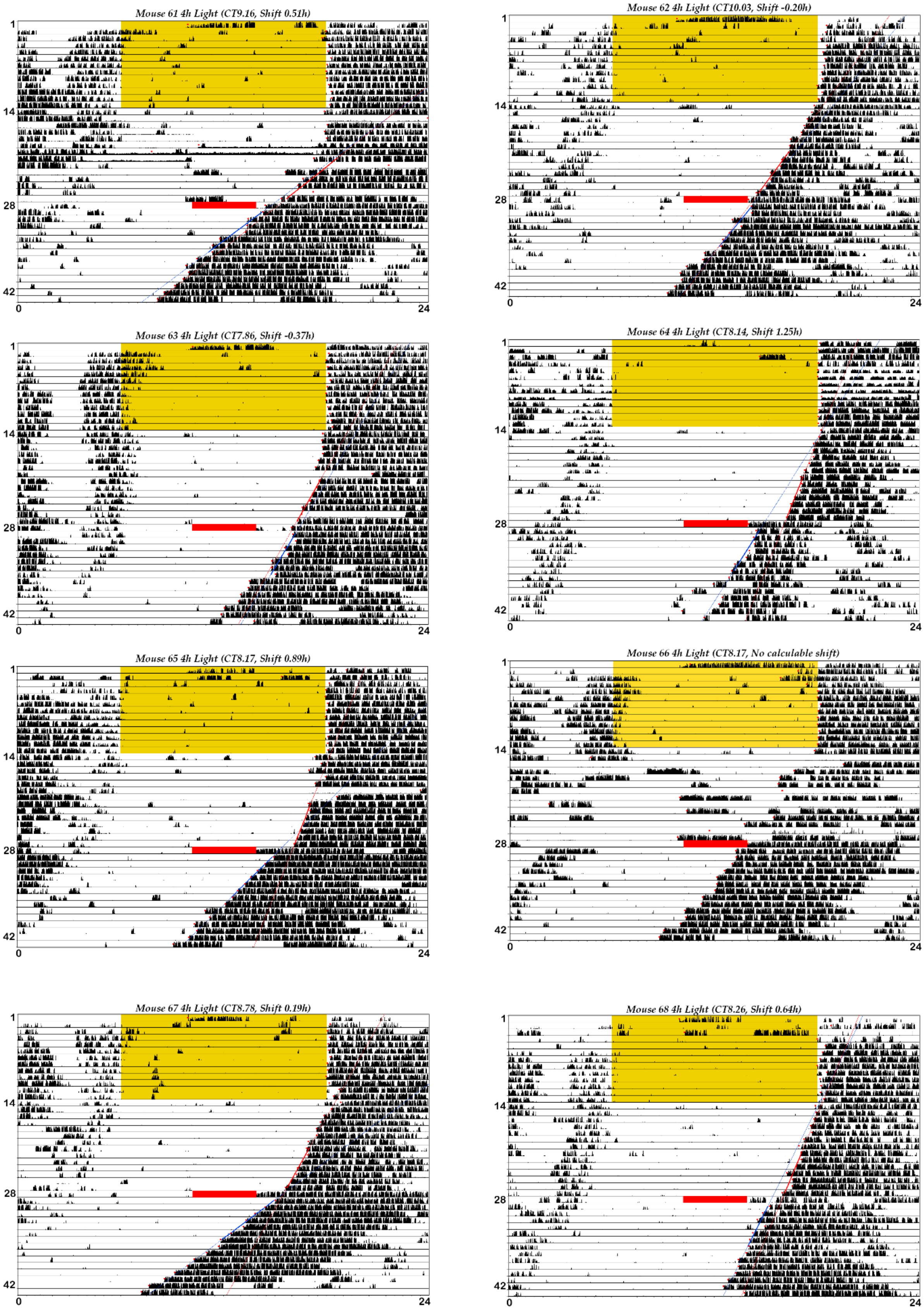

*Mouse 69 4h Light (CT9.83, Shift 0.09h)*

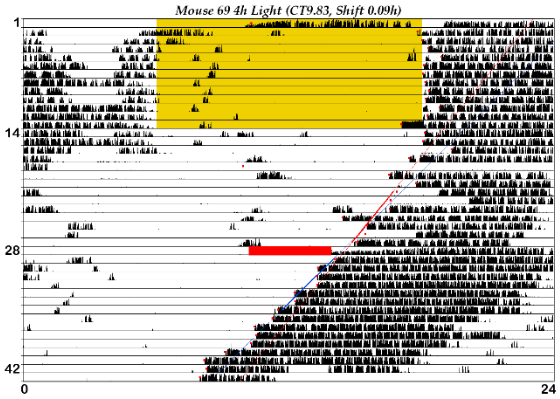


*Mouse 70 4h Light (CT8.56, Shift 0.87h)*

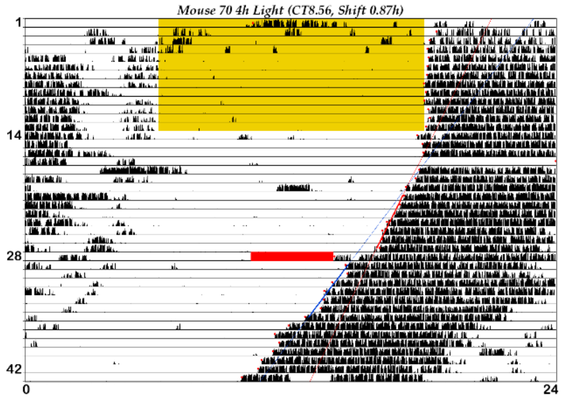


*Mouse 71 4h Light (CT2.18, Shift 0.16h)*

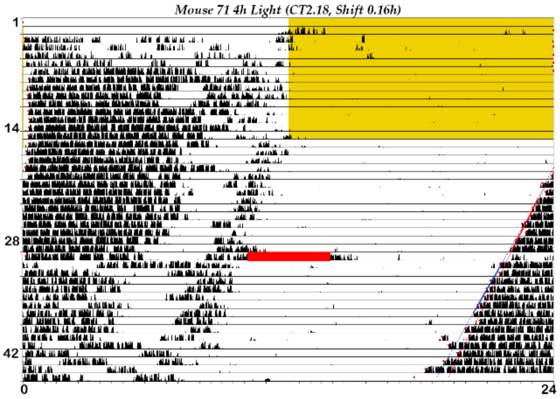


*Mouse 72 4h Light (CT0.65, Shift 1.01h)*

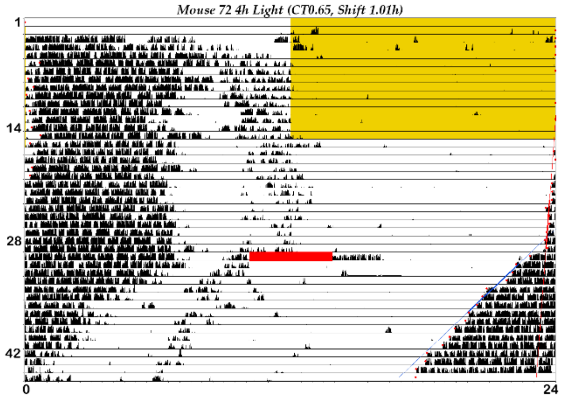


*Mouse 73 4h Light (CT2.04, Shift 0.24h)*

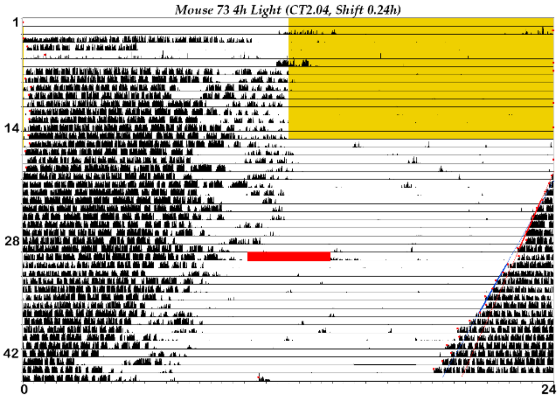


*Mouse 74 4h Light (CT1.64, Shift 0.52h)*

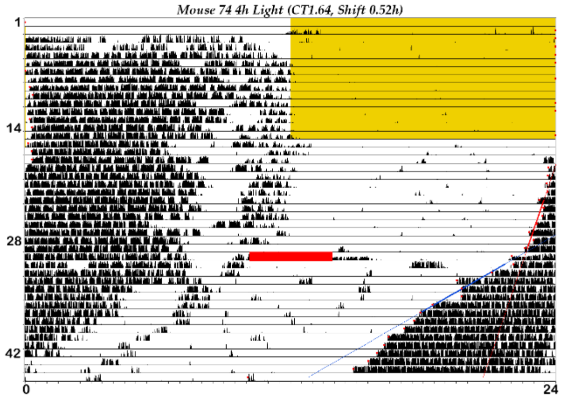


*Mouse 75 4h Light (CT1.06, Shift 0.82h)*

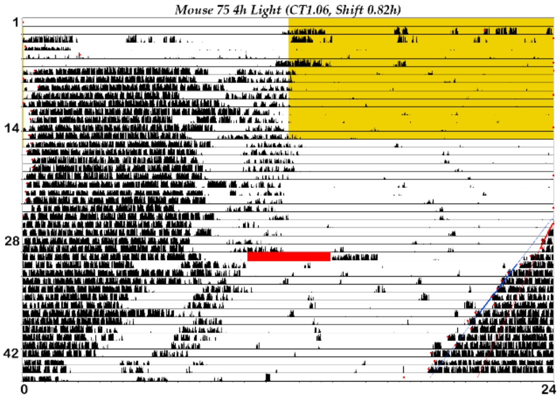


*Mouse 76 4h Light (CT3.11, Shift 0.28h)*

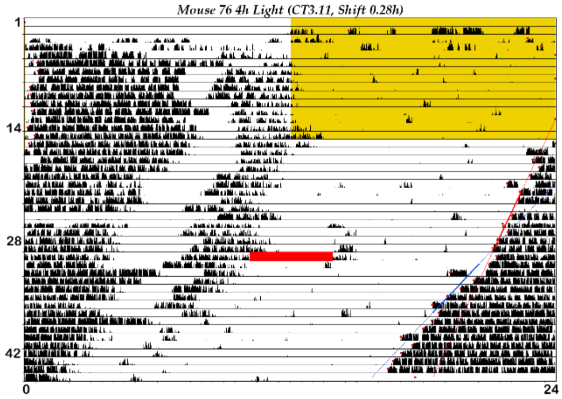

*Mouse 77 4h Light (CT3.24, Shift 0.01h)*

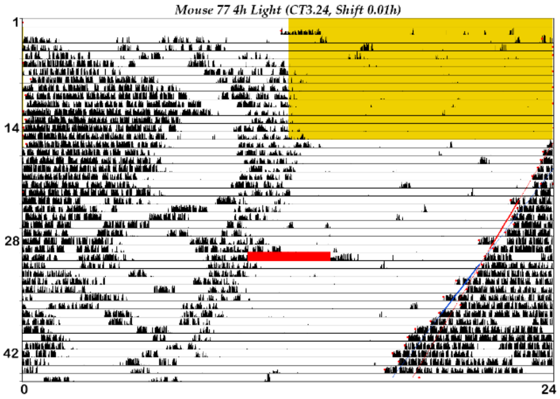


*Mouse 78 4h Light (CT3.83, Shift -0.07h)*

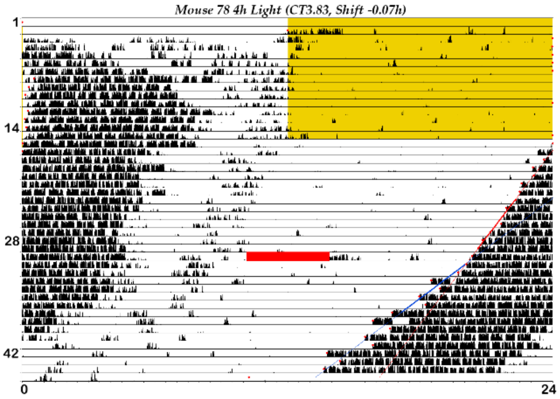


*Mouse 79 4h Light (CT2.32, Shift 0.35h)*

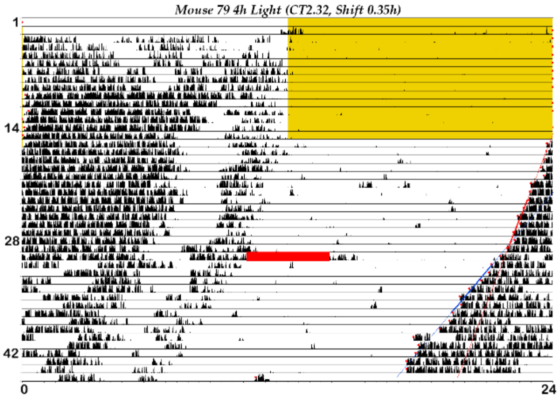


*Mouse 80 4h Light (CT1.08, Shift 0.49h)*

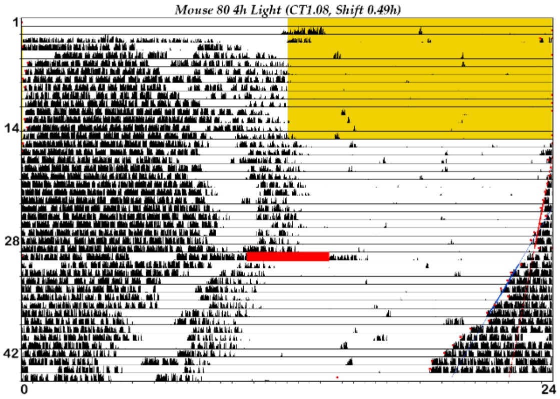


*Mouse 81 4h Light (CT4.71, Shift 0.65h)*

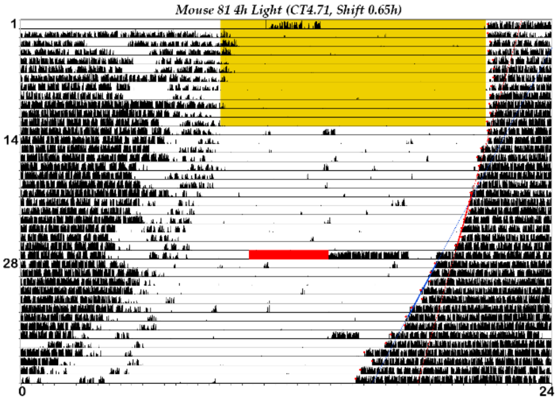


*Mouse 82 4h Light (CT4.52, Shift -0.15h)*

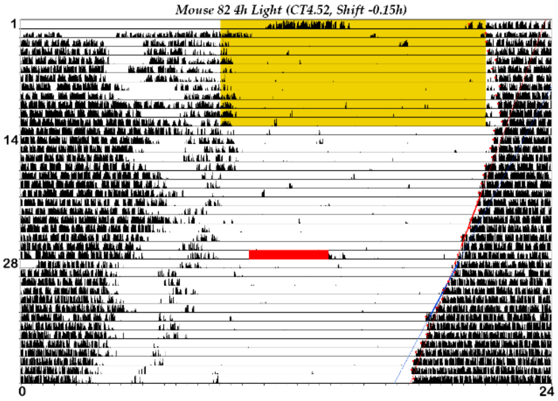


*Mouse 83 4h Light (CT4.81, Shift 0.12h)*

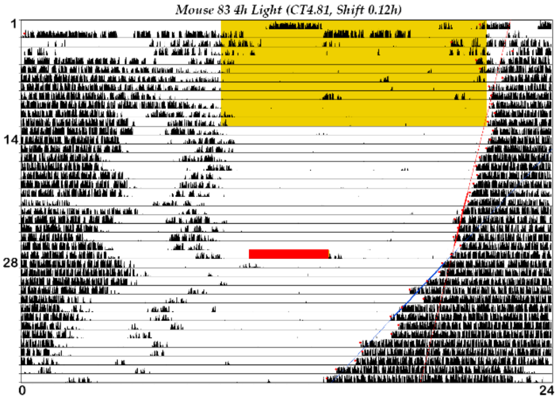


*Mouse 84 4h Light (CT6.08, Shift 1.22h)*

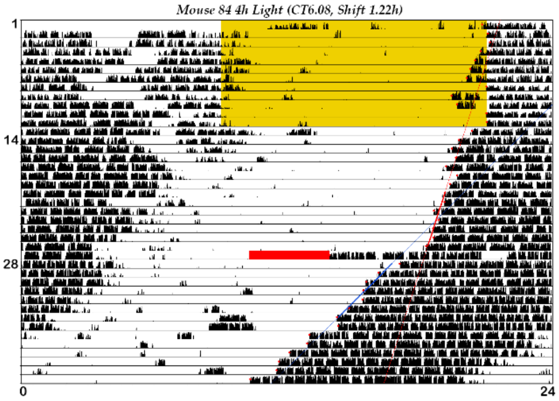

*Mouse 85 4h Light (CT5.94, Shift 0.76h)*

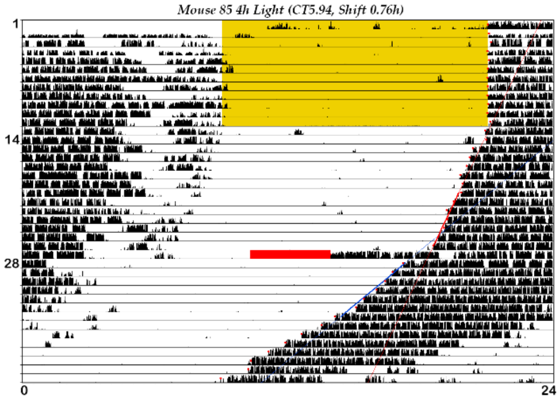


*Mouse 86 4h Light (CT6.25, Shift 0.73h)*

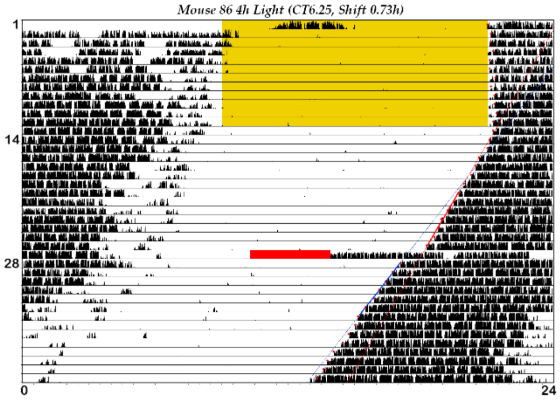


*Mouse 87 4h Light (CT6.63, Shift -0.27h)*

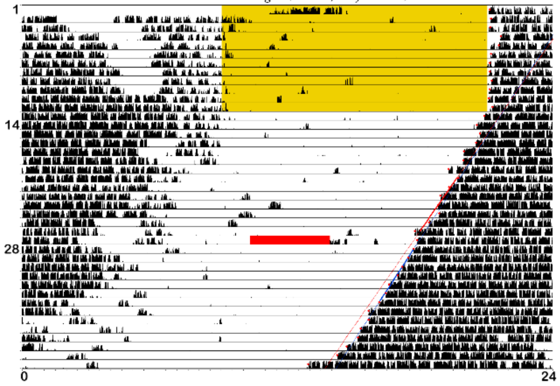


*Mouse 88 4h Light (CT7.35, Shift 0.56h)*

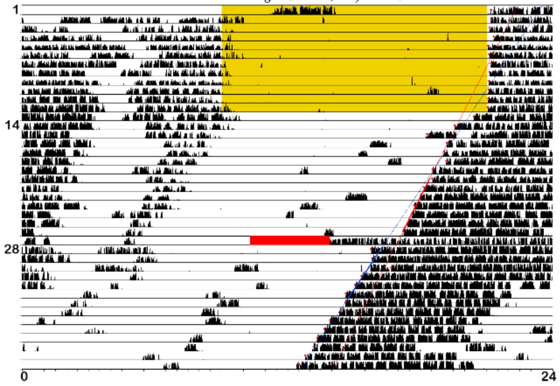


*Mouse 89 4h Light (CT6.75, Shift -0.20h)*

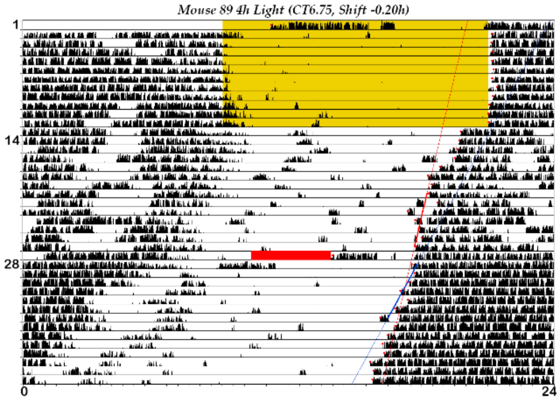


*Mouse 90 4h Light (CT4.38, Shift 0.55h)*

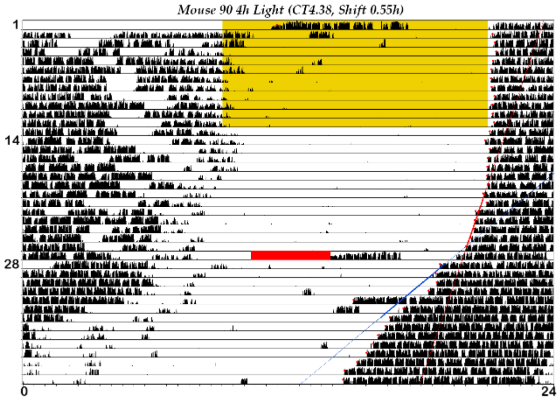


*Mouse 91 4h Light (CT22.41, Shift 0.14h)*

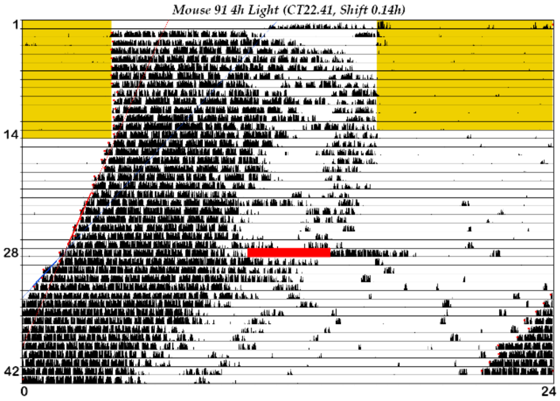


*Mouse 92 4h Light (CT18.89, Shift -1.87h)*

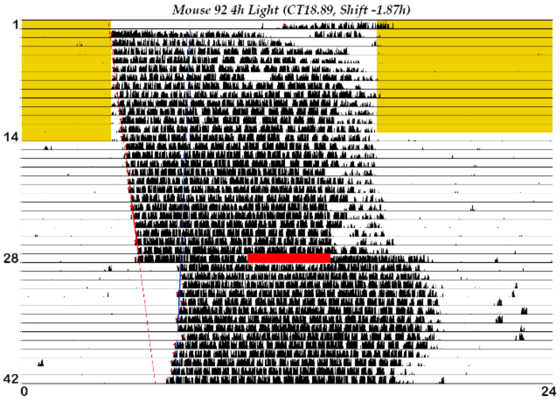

*Mouse 93 4h Light (CT22.00, Shift 0.04h)*

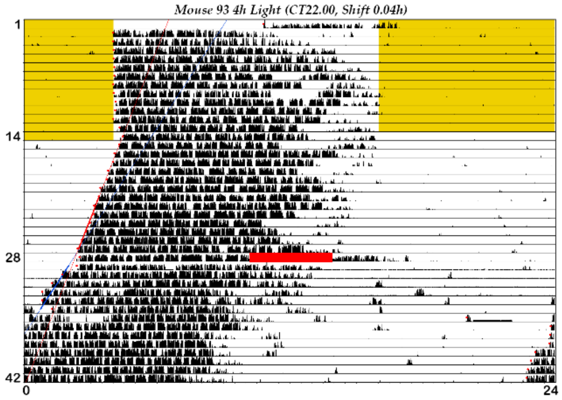


*Mouse 94 4h Light (CT20.35, Shift -5.02h)*

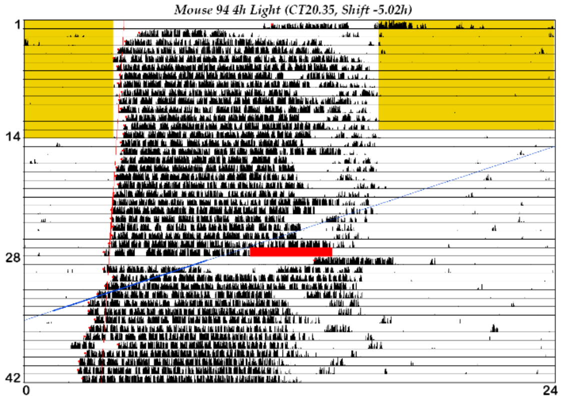


*Mouse 95 4h Light (CT20.87, Shift 0.16h)*

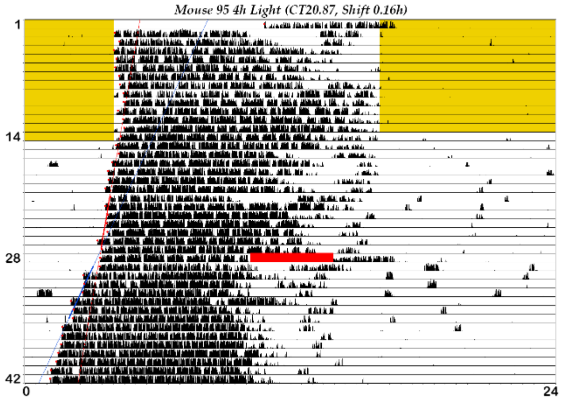


*Mouse 96 4h Light (CT21.24, Shift -1.06h)*

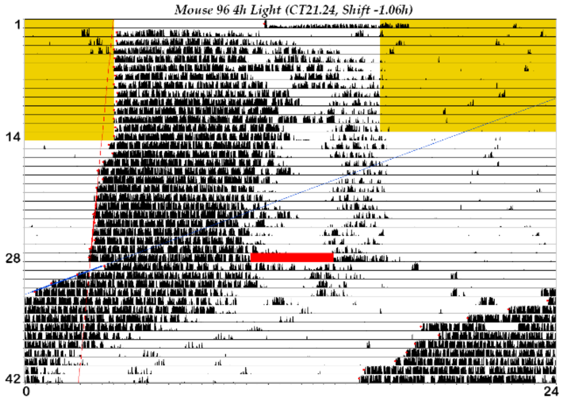


*Mouse 97 4h Light (CT0.36, Shift -0.54h)*

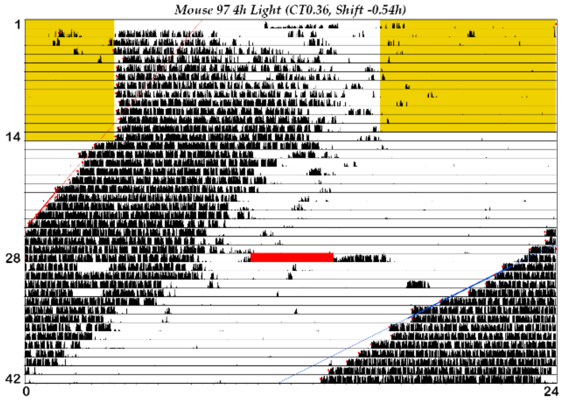


*Mouse 98 4h Light (CT19.59, Shift -4.83h)*

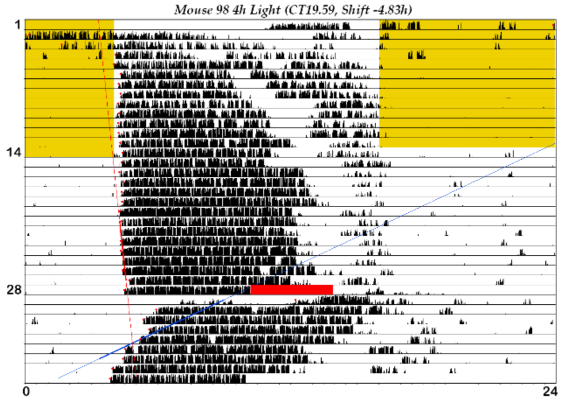


*Mouse 99 4h Light (CT20.81, Shift -0.85h)*

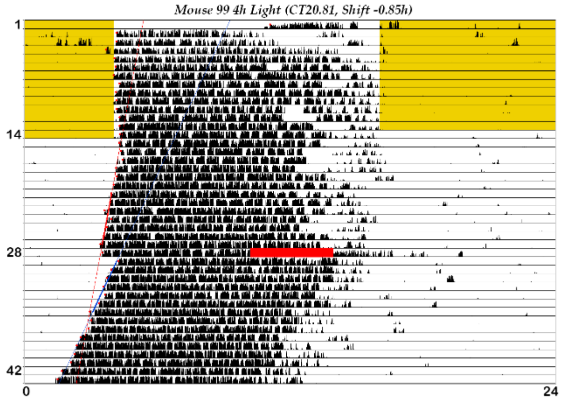


*Mouse 100 4h Light (CT20.23, Shift -0.99h)*

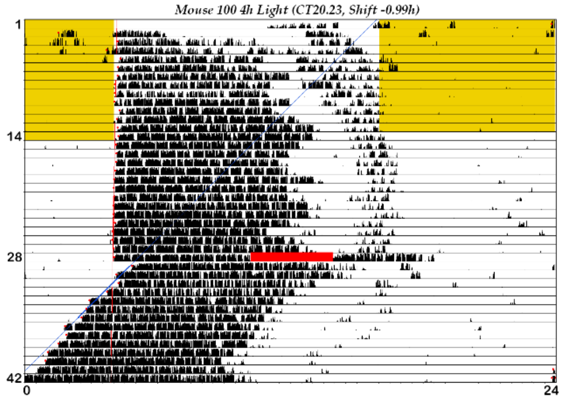

*Mouse 101 4h Light (CT16.74, Shift -4.20h)*

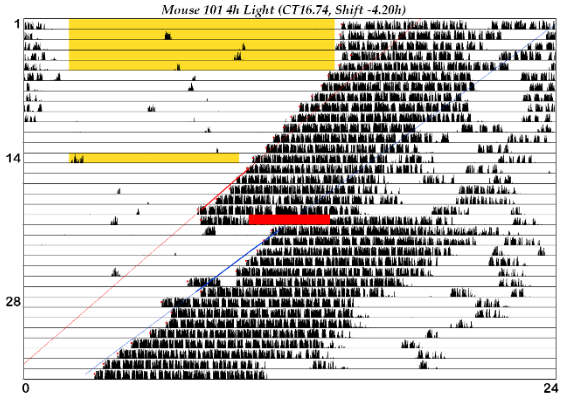


*Mouse 102 4h Light (CT15.66, Shift -3.05h)*

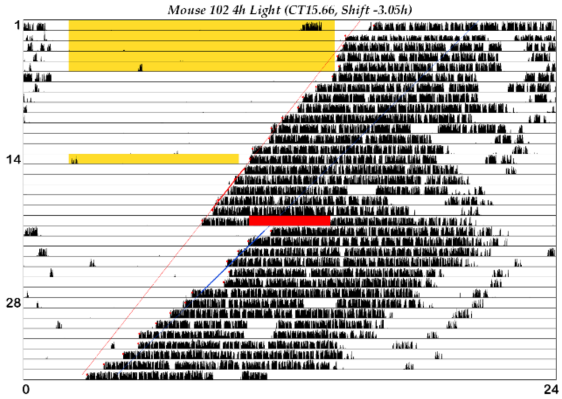


*Mouse 103 4h Light (CT18.02, Shift -3.54h)*

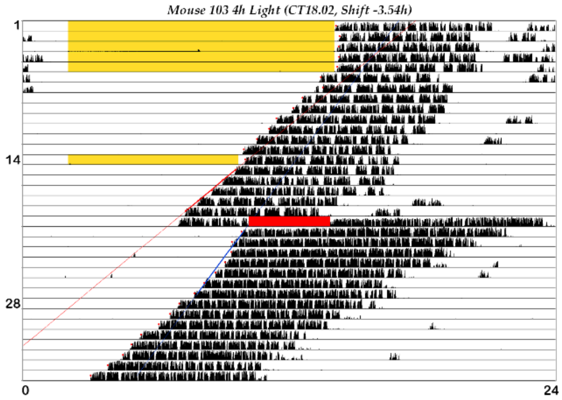


*Mouse 104 4h Light (CT15.81, Shift -4.57h)*

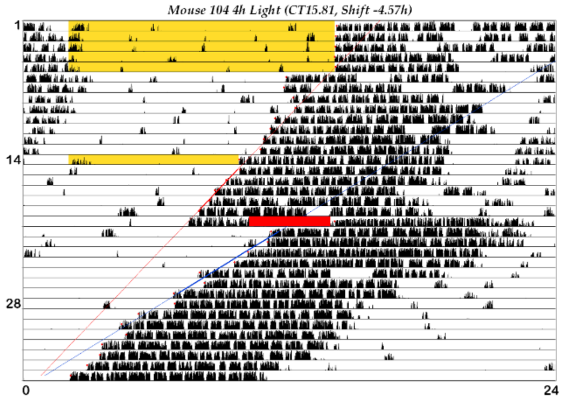


*Mouse 105 4h Light (CT16.87, Shift -3.61h)*

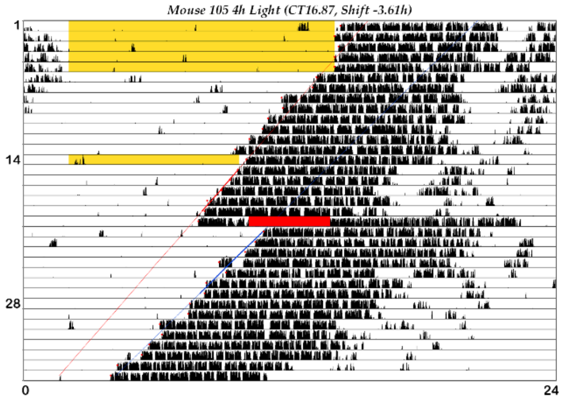


*Mouse 106 4h Light (CT16.34, Shift -4.19h)*

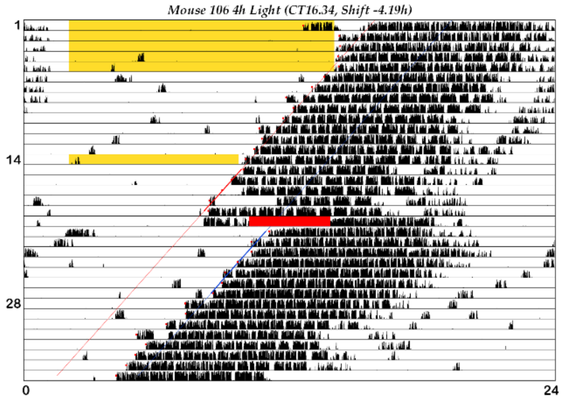


*Mouse 107 4h Light (CT15.07, Shift -3.22h)*

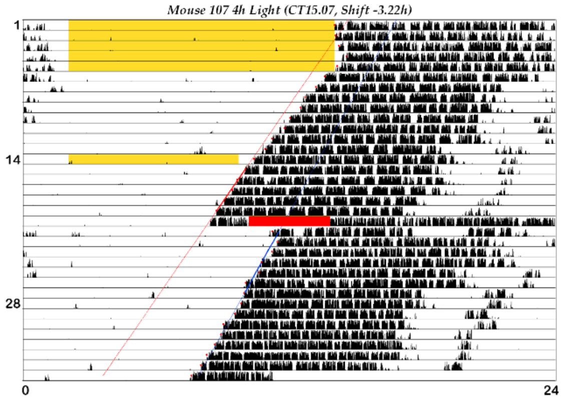


*Mouse 108 4h Light (CT16.44, Shift -3.33h)*

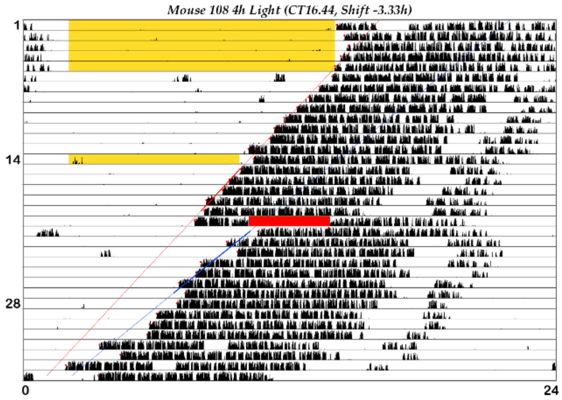

*Mouse 109 4h Light (CT16.82, Shift -2.91h)*

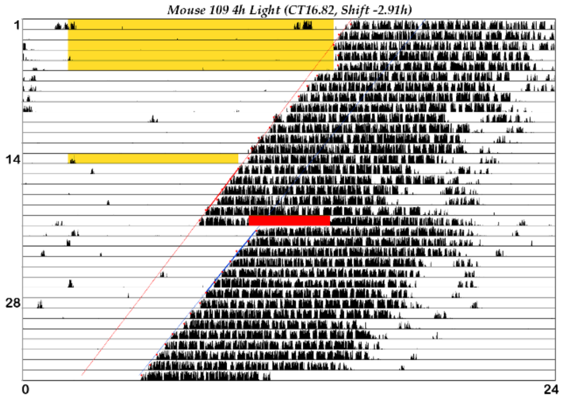


*Mouse 110 4h Light (CT16.89, Shift -3.23h)*

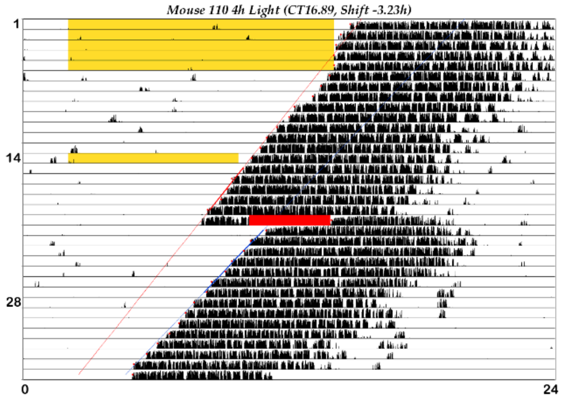


*Mouse 111 4h Light (CT17.65, Shift -3.13h)*

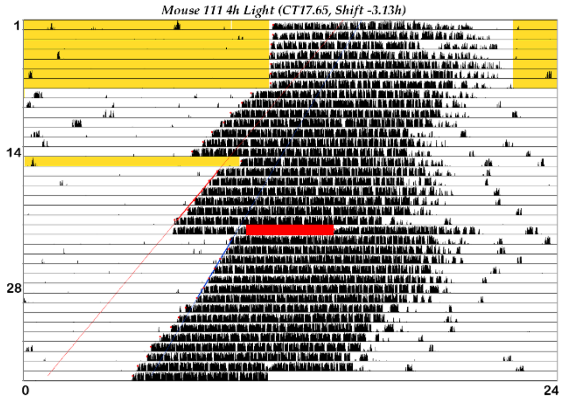


*Mouse 112 4h Light (CT14.67, Shift -2.55h)*

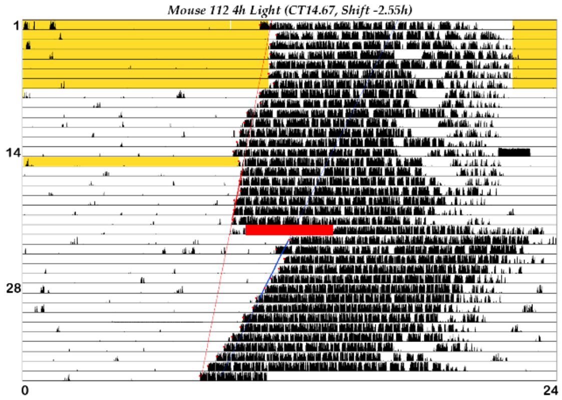


*Mouse 113 4h Light (CT16.68, Shift -2.75h)*

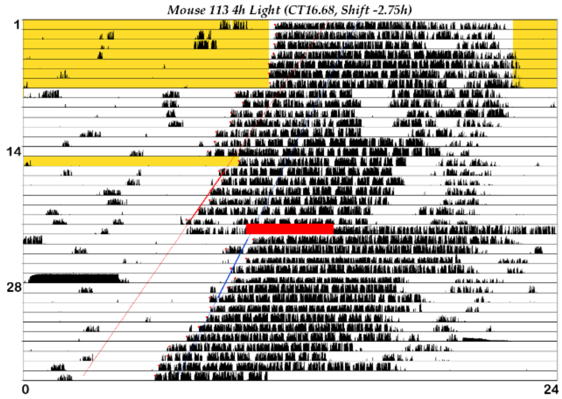


*Mouse 114 4h Light (CT14.94, Shift -3.05h)*

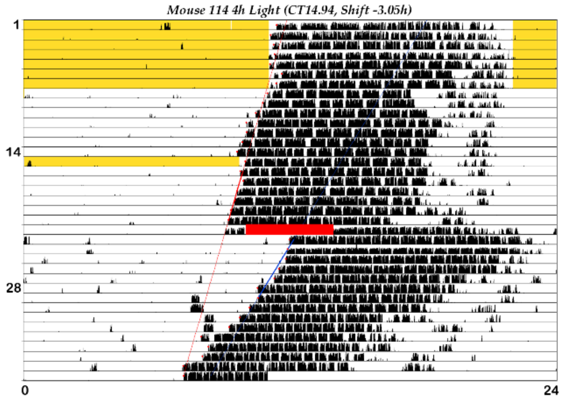


*Mouse 115 4h Light (CT16.86, Shift -2.78h)*

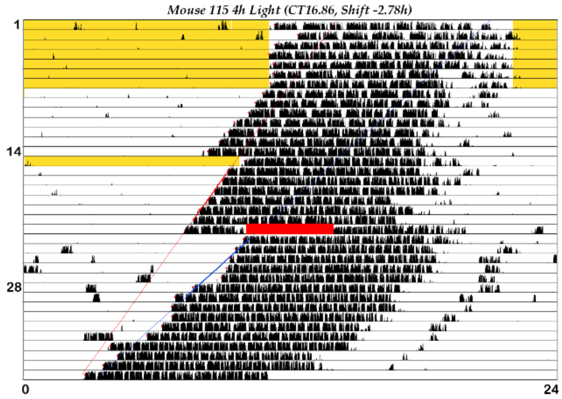


*Mouse 116 4h Light (CT16.05, Shift -3.36h)*

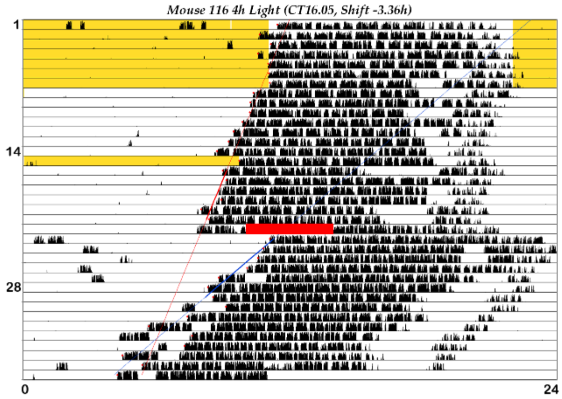

*Mouse 117 4h Light (CT-, No calculable shift)*

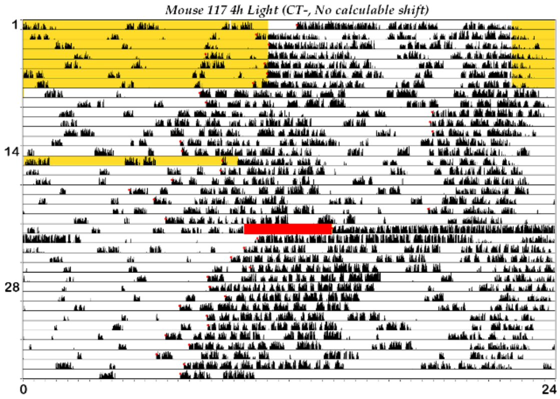


*Mouse 118 4h Light (CT18.58, Shift -1.85h)*

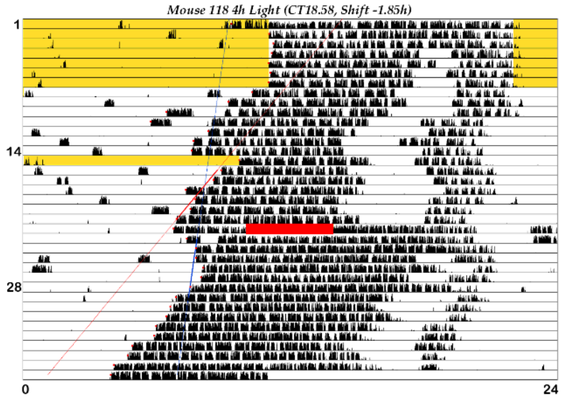


*Mouse 119 4h Light (CT18.03, Shift -3.48h)*

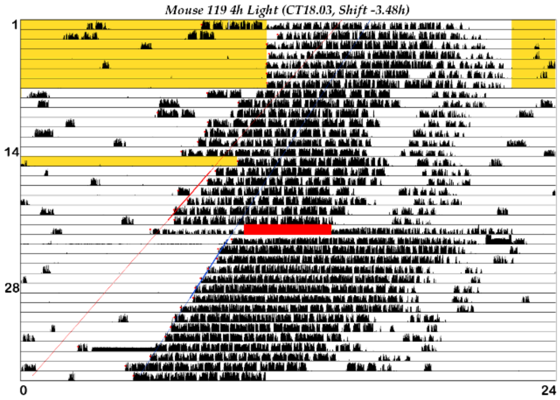


*Mouse 120 4h Light (CT15.94, Shift -2.86h)*

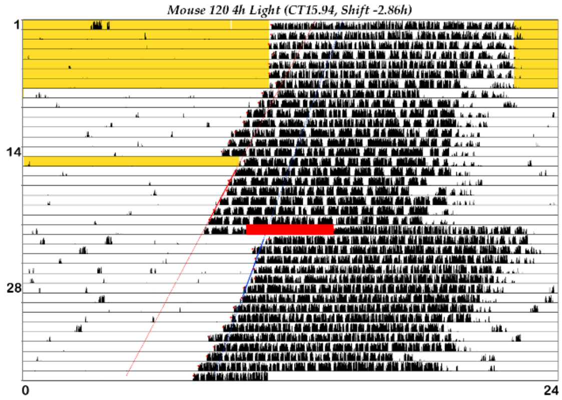

Supplement: Supplementary file 1 [file clockssleep-03-00034-s001.zip › Supplementary Figure S2.pdf]
