# Supplementary material for: The Effects of General Anaesthesia and Light on Behavioural Rhythms and GABAA Receptor Subunit Expression in the Mouse SCN
Source: Clocks Sleep. 2021 Sep 17;3(3):482–94. doi: 10.3390/clockssleep3030034 (PMC8482144; doi:10.3390/clockssleep3030034)
Supplement: Supplementary file 1 [file clockssleep-03-00034-s001.zip › Supplementary Table S1.pdf]

Table S1: Table of Behavioural Phase shifts of C57 BL6/VJU mice exposed to GA (isoflurane) + Light

Animal #	CT	GA Shift (h)
1	11.98	-1.5
2	7.92	-0.31
3	9.50	-0.29
4	8.97	-0.07
5	9.69	-0.68
6	10.60	-0.6
7	8.50	0.14
8	8.92	-0.25
9	8.53	0.6
10	8.02	-4.7
11	3.34	-0.35
12	23.91	0.05
13	3.03	0.2
14	2.59	-0.27
15	23.75	0.63
16	23.4	0.66
17	23.84	0.33
18	0.58	0.48
19	2.40	-0.27
20	1.54	0.94
21	4.67	-1.75
22	6.81	-0.33
23	6.38	0.15
24	7.82	0.12
25	5.24	0.63
26	3.22	-0.07
27	5.17	-0.39
28	4.05	0.54
29	6.69	0.01
30	6.70	-0.24
31	23.97	0.32
32	21.60	0.21
33	22.98	0.62
34	22.87	-0.24
35	21.34	0.17
36	22.03	-0.7
37	24	0
38	21.11	0.51
39	19.62	0.66
40	22.35	-0.25
41	15.51	-1.18
42	17.15	-0.6
43	14.17	-2.41
44	16.68	-0.67
45	17.20	-1.21
46	16.89	0.24
47	17.66	-1.07
48	16.97	-1.89
49	20.83	0.93
50	14.36	-2.7

51	16.78	0.61
52	15.79	-2.05
53	18.20	0.41
54	15.27	-1.69
55	16.84	-0.97
56	16.50	-0.14
57	14.97	-1.17
58	16.64	-0.04
59	16.87	-0.68
60	17.03	-1
