# Supplementary material for: The Effects of General Anaesthesia and Light on Behavioural Rhythms and GABAA Receptor Subunit Expression in the Mouse SCN
Source: Clocks Sleep. 2021 Sep 17;3(3):482–94. doi: 10.3390/clockssleep3030034 (PMC8482144; doi:10.3390/clockssleep3030034)
Supplement: Supplementary file 1 [file clockssleep-03-00034-s001.zip › Supplementary Table S2.pdf]

Table S2: Table of Behavioural Phase shifts of C57 BL6/VJU mice exposed to light

Animal #	CT	GA Shift (h)
61	9.16	0.51
62	10.03	-0.2
63	7.86	-0.37
64	8.14	1.25
65	8.17	0.89
66	-	-
67	8.78	0.19
68	8.26	0.64
69	9.83	0.09
70	8.56	0.87
71	2.18	0.16
72	0.65	1.01
73	2.04	0.24
74	1.64	0.52
75	1.06	0.82
76	3.11	0.28
77	3.24	0.01
78	3.83	-0.07
79	2.32	0.35
80	1.08	0.49
81	4.71	0.65
82	4.52	-0.15
83	4.81	0.12
84	6.08	1.22
85	5.94	0.76
86	6.25	0.73
87	6.63	-0.27
88	7.35	0.56
89	6.75	-0.2
90	4.38	0.55
91	22.41	0.14
92	18.89	-1.87
93	22.00	0.04
94	20.35	-5.02
95	20.87	0.16
96	21.24	-1.06
97	0.36	-0.54
98	19.59	-4.83
99	20.81	-0.85
100	20.23	-0.99
101	16.74	-4.2
102	15.66	-3.05
103	18.02	-3.54
104	15.18	-4.57
105	16.87	-3.61
106	16.34	-4.19
107	15.07	-3.22
108	16.44	-3.33
109	16.82	-2.91
110	16.89	-3.23

111	17.65	-3.13
112	14.67	-2.55
113	16.68	-2.75
114	14.94	-3.05
115	16.86	-2.78
116	16.05	-3.36
117	-	-
118	18.58	-1.85
119	18.04	-3.48
120	15.94	-2.86
